# Honing-in antigen-specific cells during antibody discovery: a user-friendly process to mine a deeper repertoire

**DOI:** 10.1038/s42003-022-04129-7

**Published:** 2022-10-30

**Authors:** Ankit Mahendra, Aftabul Haque, Ponraj Prabakaran, Brian C. Mackness, Thomas P. Fuller, Xiaohua Liu, Sagar V. Kathuria, Yui-Hsi Wang, Nilesh Amatya, Xiaocong Yu, Joern Hopke, Dietmar Hoffmann, Eva Bric-Furlong, Ningning Zhang, Hyun-Suk Cho, Ruijun Zhang, Jose Sancho, Jacqueline Saleh, Sambasiva P. Rao, Maria Wendt, Partha S. Chowdhury

**Affiliations:** 1 Large Molecule Research, Sanofi, 49 New York Avenue, Framingham, MA USA; 2 Immunology & Inflammation Research, Genomics Center, Sanofi, 270 Albany Street, Cambridge, MA USA; 3 Molecular Expression and Screening Technologies, Sanofi, 270 Albany Street, Cambridge, MA USA; 4 Neuroinflammation, Sanofi Genzyme, 49 New York Avenue, Framingham, MA USA; 5 Present Address: Johnson & Johnson R&D Center, 1400 McKean Road, Spring House, PA 19477 USA

**Keywords:** Antibody therapy, Antibody generation

## Abstract

Immunization based antibody discovery is plagued by the paucity of antigen-specific B cells. Identifying these cells is akin to finding needle in a haystack. Current and emerging technologies while effective, are limited in terms of capturing the antigen-specific repertoire. We report on the bulk purification of antigen-specific B-cells and the benefits it offers to various antibody discovery platforms. Using five different antigens, we show hit rates of 51–88%, compared to about 5% with conventional methods. We also show that this purification is highly efficient with loss of only about 2% antigen specific cells. Furthermore, we compared clones in which cognate chains are preserved with those from display libraries in which chains either from total B cells (TBC) or antigen-specific B cells (AgSC) underwent combinatorial pairing. We found that cognate chain paired clones and combinatorial clones from AgSC library had higher frequency of functional clones and showed greater diversity in sequence and paratope compared to clones from the TBC library. This antigen-specific B-cell selection technique exemplifies a process improvement with reduced cycle time and cost, by removing undesired clones prior to screening and increasing the chance of capturing desirable and rare functional clones in the repertoire.

## Abstract (web summary)

The authors developed a methodology for bulk enrichment of antigen-specific B cells to improve the diversity of antibody sequences and increase the number of functional antigen-specific antibodies coming from different antibody generation platforms.

## Introduction

Monoclonal antibodies as a promising therapeutic modality are now widely accepted. As of now there are 129 antibody therapeutics approved or under review in the US and EU^1,2^. The revelation from Dennis Burton’s group that antibodies derived by B-cell cloning had greater therapeutic potency compared to those derived from combinatorial libraries from HIV convalescent patients^3^, led to a surge in efforts to discover therapeutic antibodies from immunized animals or convalescent patients^4–12^. However, antibody discovery from immunized animals or convalescent patients, is an intricate process due to the scarcity of antigen-specific cells in a vast diversity of the overall antibody repertoire. The frequency of antigen-specific cells, even after a robust immune reaction, is only between 0.01–0.1% of total B cells^13–15^. This creates a formidable challenge even for the most advanced screening platforms to mine the entire immune repertoire. Therefore, platforms that rely on immunization of animals, or use of convalescent patient B cells, deal with a huge abundance of irrelevant cells, leading to high attrition between screening and identification of antigen-specific hits. A popular approach to deep-screen immune repertoire is to make immunized phage libraries, several of which have been reported to be a good source of potent antibodies^16–18^. However, during construction of immune display libraries, cognate chain pairs from the small percentage of antigen-specific cells undergo extensive combinatorial pairing with the vast repertoire of chains from non-antigen-specific cells^19^. Despite such combinatorial pairings, parental cognate pairing is probably retained at a low frequency^18^. We hypothesized that increasing the frequency of the parental cognate chain pairings by constructing and panning immunized library generated from antigen-specific B cells (AgSC), could possibly provide greater success in mining the antigen-specific repertoire.

Antigen-specific B cells are commonly isolated by fluorescent activated cell sorting (FACS)^6–12,20,21^. While flow cytometry works well for single B-cell cloning, its use for bulk isolation is limited by its speed and the harsh effect that electromagnetic field can have on cell integrity at high sorting speed^22^, leading to significant loss of cell quantity and quality^23^. Therefore, mild yet efficient methods to isolate the AgSC from immunized animals are needed to interrogate the immune repertoire deep and wide. We have developed a simple yet powerful antigen-specific memory B-cell (MBC) bulk selection technique for rapid and effective identification of monoclonal IgGs from immunized mice. This technique is based on the fact that MBCs express surface IgGs, and magnetic nanoparticle assisted cell sorting (MACS) can be employed to select antigen-specific MBCs quickly under mild conditions by using biotinylated antigens.

After bulk isolation of the AgSCs, label-free cell isolation combined with single-cell sorting, efficient V-domain cloning, and high throughput IgG expression^21^, we demonstrated that AgSCs provide substantial enrichment leading to 51–88% target-specific monoclonal antibodies. This process is highly efficient, as only a tiny fraction of antigen-specific cells is left uncaptured after the selection steps. We validated our approach by generating antigen-specific recombinant monoclonal IgGs from mice within two weeks post immunization for five target antigens. A similar AgSC enrichment approach has recently been reported^24^; however, this study does not offer an insight into how the method can aid in monoclonal generation through different platforms.

In this work, we report a standalone technique to quickly obtain cognate chain paired antigen-specific monoclonal antibodies for five targets, by bulk isolation of AgSCs from immunized animals. For 2 of the 5 targets, we report a comparative study of phage-display libraries made by combinatorial chain pairing within populations of AgSCs, and IgG^+^ total B cells (TBC). Through a comprehensive V-gene analysis we identify that the AgSC libraries when compared to the TBC libraries generated from the same animals, demonstrated much higher frequency of sequence and chain-pairing diversity, showed a greater number of paratope clusters and most importantly yielded higher number of functional clones, all of which are highly desirable features of antibodies in discovery campaigns. We show that in terms of function, the cognate chain paired clones offer the best functional activity, followed by clones rescued from AgSC library and least from TBC library. Through this comparative approach, this study re-enforces the importance of cognate chain pairing of antibodies from immunized sources and suggests its application in emerging platforms such as, microfluidics-based antibody discovery^25–27^, which are still plagued by the scarcity of antigen specific cells.

## Results

### Bulk-selection of antigen-specific B cells from immunized mice

**Fig. 1:**
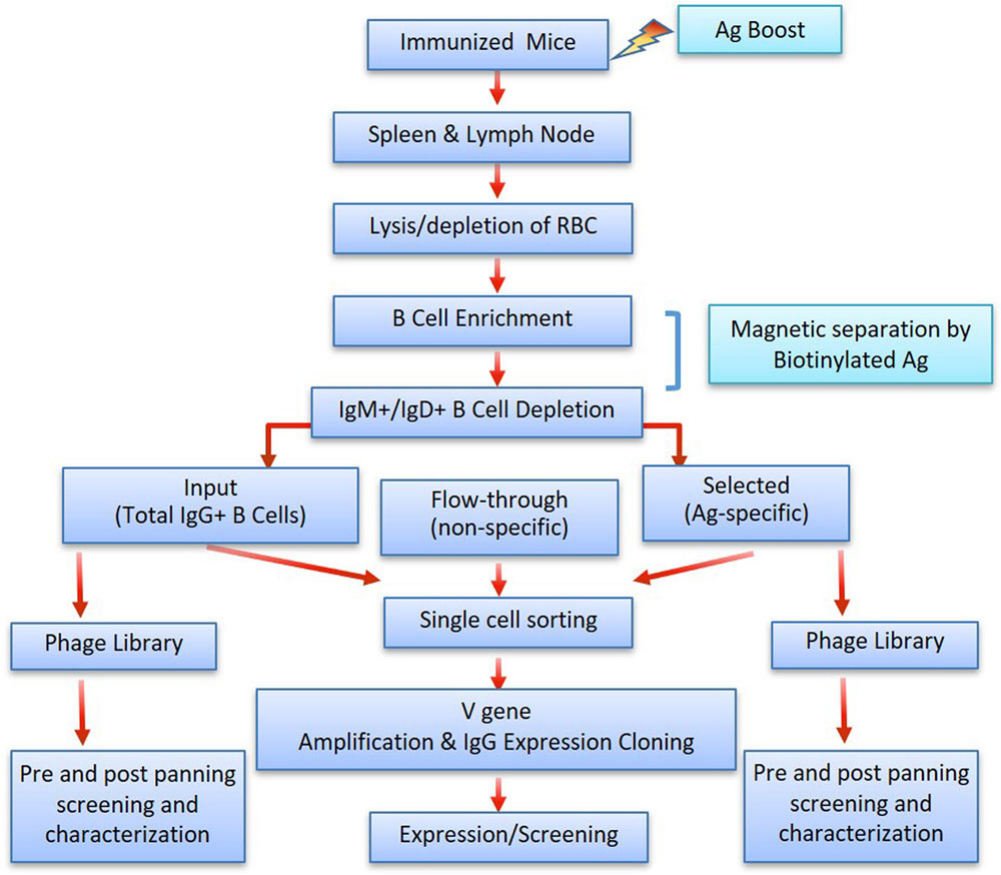
Flowchart of the study depicting antigen-specific B-cell bulk selection and their characterization by single cell cloning, and phage library construction. Cell suspension was prepared from spleen and lymph nodes and total B-cell was enriched by RBC lysis followed by immuno-depletion of non-B cells by negative selection. IgG^+^ B cells were enriched by depleting IgM^+^ and IgD^+^ B cells. Antigen-specific B cells were isolated by using biotinylated target antigen in solution. Input, selected and flow-through B-cell populations were single-sorted by FACS in 96-well plate. From single B cells, cDNA was prepared, and variable regions of individual IgG genes were amplified. Heavy and kappa chain expression cloning were performed for each sorted B-cell and co-expressed in Expi293F cells to generate recombinant antibodies for antibody identification and method validation.

**Fig. 2:**
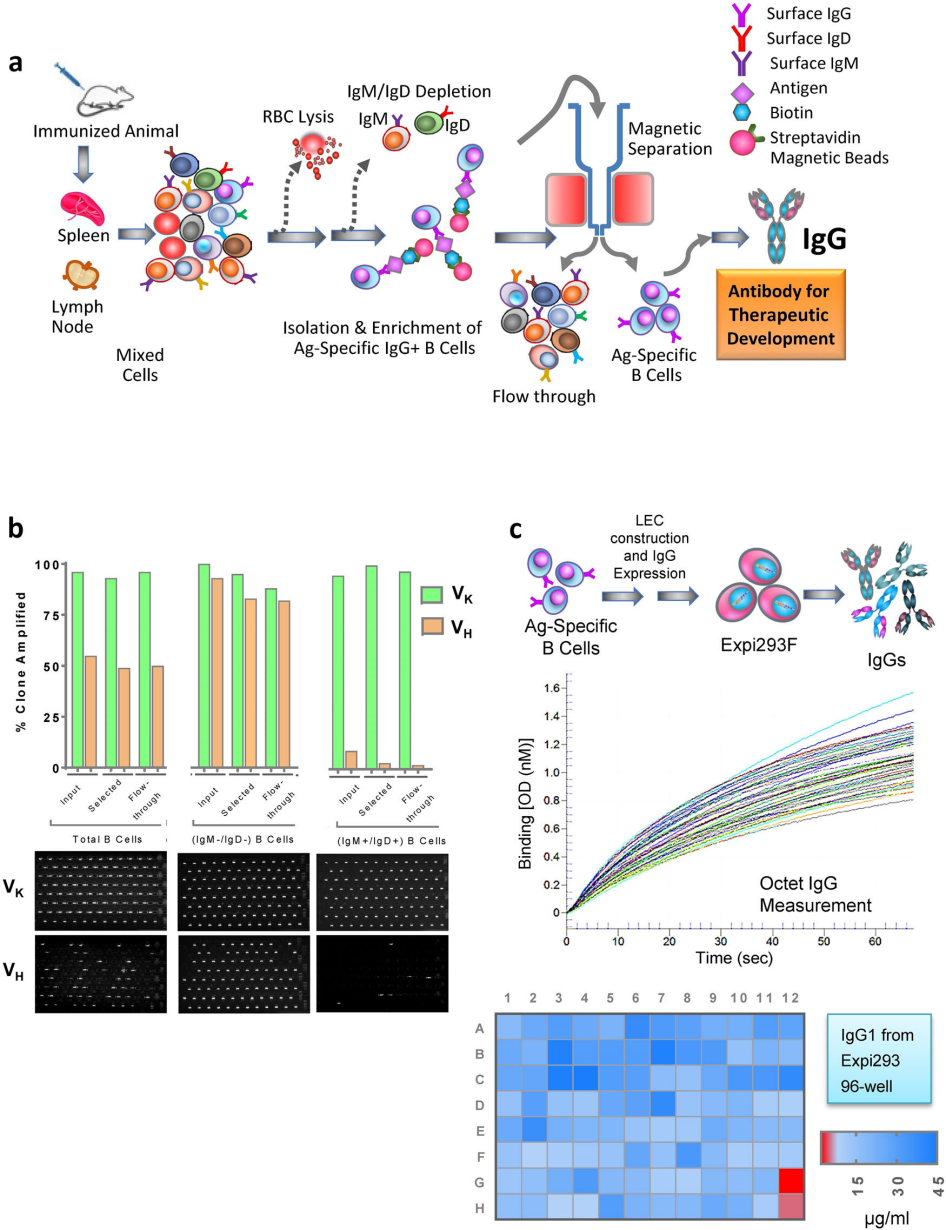
Ag-specific B-cell selection in bulk, V-gene recovery from single B cells, expression and quantitation of r-IgG. **a** A schematic diagram shows antigen-specific B-cell selection and isolation from mice immunized with a target antigen. IgG^+^ B cells (cell surface) were enriched by depletion of non-B cells and IgM^+^ and IgD^+^ (cell surface). Biotinylated antigen was added in solution and target-specific IgG^+^ B cells were isolated by magnetic separation. Viable Ag-specific single B cells were sorted in each well of a 96-well plate. Single B-cell expression cloning was performed to generate rIgG heavy and kappa chains. **b** Recovery of paired V_H_ and Vκ was determined by eGel analysis of amplified DNA. Bar graph shows percent of clones, which were amplified to yield V_K_ and V_H_ genes from single B cells. Representative eGel analysis of amplified V_H_ and VK genes are shown at the bottom for total B cells, IgG^+^ B-cells (after IgM^+^/IgD^+^ cells were depleted) and IgM^+^/IgD^+^ B cells. **c** Schematic representation of expression of individual rIgGs recovered from single antigen-selected B cells. Paired rIgG heavy and kappa chains were co-expressed in Expi293F cells for transient expression of individual antibodies. Antibody concentrations in conditioned media from transfected Expi293F cultures was measured by Octet Red96 (ForteBio). Representative quantitation of 94 IgG samples in Expi293F conditioned media is shown in the graph. A representative heatmap demonstrates the range of individual rIgG expression from Expi293F cells in each well of a 96-well culture plate, 6 days post-transfection.

Transgenic mice with human variable region repertoires (Trianni, San Francisco, USA) were immunized with different antigenic proteins of therapeutic importance anonymized as A, B, C, D and E for 4–5 times, two weeks apart. All these targets are immunomodulatory in nature, wherein, antigens A, B, and E are expressed both as surface bound to cells and soluble in circulation, antigen C is the ectodomain of a cell-surface receptor, and antigen D a cytokine expressed as a soluble protein in circulation. Spleen and lymph nodes were harvested for selecting Ag-specific B-cells. Isolation of Ag-specific B-cells was performed as depicted in Fig. 1. We first isolated B cells from spleen and lymph nodes using a B-cell enrichment kit, and further depleted IgM^+^/IgD^+^ B cells to enrich IgG^+^ B cells, which were then used for antigen specific selection (Fig. 2a). For capturing antigen-specific B cells, we used Avi-tagged antigens that were biotinylated in a cellular system co-expressing biotin ligase along with the recombinant protein of interest. This in vivo biotinylation adds biotin site-specifically to the Avi tag^28,29^ and is expected to cause no change in antigen function, thus suitable for screening functional antibodies. Bulk selection of antigen-specific IgG^+^ B cells (AgSCs) was performed by using a biotinylated target antigen in the presence of non-biotinylated irrelevant antigen to exclude tag-specific B cells. We observed that the selected B cells were ~95% viable (Table 1), indicating that the steps involved are not detrimental to cellular health. The antigen specificity of magnetically selected AgSCs was evaluated by isolating individual IgGs from single B cells by V-gene cloning in 96 well plates. Table 1 also shows that a small fraction of antigen-specific cells for each of the targets (0.19–1.12% of B cells) were isolated after antigen selection and ~95% or more of the starting B cells remain in the flow-through fractions.

**Table 1:** Relative number of cells at different stages of antigen specific B-cell isolation process for 5 antigens (A-E). The starting material were pools of cells from spleen and lymph nodes from 3 animals for each study.

Target antigen	Splenocytes (x10^6^)	B cells (x10^6^)	Total IgG^+^ B cells (x10^6^)	Antigen-selected B Cells (x10^6^)	Flow through cells (x10^6^)	% Viability of antigen-selected B cells	% Antigen-selected Cells (of total B Cells)	% Antigen-selected Cells (of IgG^+^ B Cells)
A	304	67	19	0.13	18.3	94.5	0.19	0.66
B	301	70	22	0.15	20.0	95.3	0.22	0.71
C	285	75	25	0.72	23.7	96.7	0.95	2.92
D	304	79	27	0.88	26.4	95.5	1.12	3.25
E	294	83	24	0.21	23.1	94.7	0.25	0.87

### Efficient IgG gene recovery and expression was achieved from single B cells

We produced recombinant IgGs (rIgG) from the three B-cell populations, input, selected and the flow-through (Fig. 2b). The input IgGs represent individual antibodies recovered from single cells of the total IgG^+^ B cells without any target-specific enrichment selection. The selected IgGs represent individual antibodies isolated from single B cells expressing surface IgGs specific to a population selected with the target antigen. The third group, flow-through IgGs represent antibodies recovered from single B-cells that were not selected by the target antigen. Live B-cells gated after 7-ADD staining for dead cells were sorted at 1 cell/well into 96-well PCR plates containing lysis buffer and RT reaction components. Total mRNA from the individual cells was extracted and cDNA was synthesized. Paired IgG genes (V_H_ and V_K_ chains) from individual B cells were amplified, as described previously^21^. Recovery of V_H_ and V_K_ genes from the single-sorted B cells was analyzed using 96-well eGel (Fig. 2b). We observed efficient recovery of paired V_H_ and V_K_ IgG genes from single B cells for all 5 antigens, A (86%), B (81%), C (95%), D (94%), E (99%) (Fig. 2b). These recovered V-genes were then expressed as IgGs by transfecting Expi293F cells in suspension culture with linear expression cassettes (Fig. 2c) in a high throughput format^21^.

IgG expression was sufficient to be quantitated by Octet (Fig. 2c) and test for antigen binding (8–45 µg/ml). The supernatant of mock transfected Expi293F cells did not show any reactivity in the Octet measurement. These results demonstrated that our approach for IgG gene recovery and high throughput expression worked efficiently.

### Magnetic bulk selection of B cells by antigen-coated beads resulted in a highly enriched population of antigen-specific B cells

To investigate whether the antigen specific B-cell selection method really leads to enrichment of clones with the desired specificity, we investigated antigen binding of the individual antibodies from three B-cell populations - input, selected and flow-through. For each of the five target antigens, 288 individual IgGs from the antigen-selected group and 96 individual IgGs from the input and flow-through B-cell groups were expressed and tested for gene recovery, expression, and target binding.

**Fig. 3:**
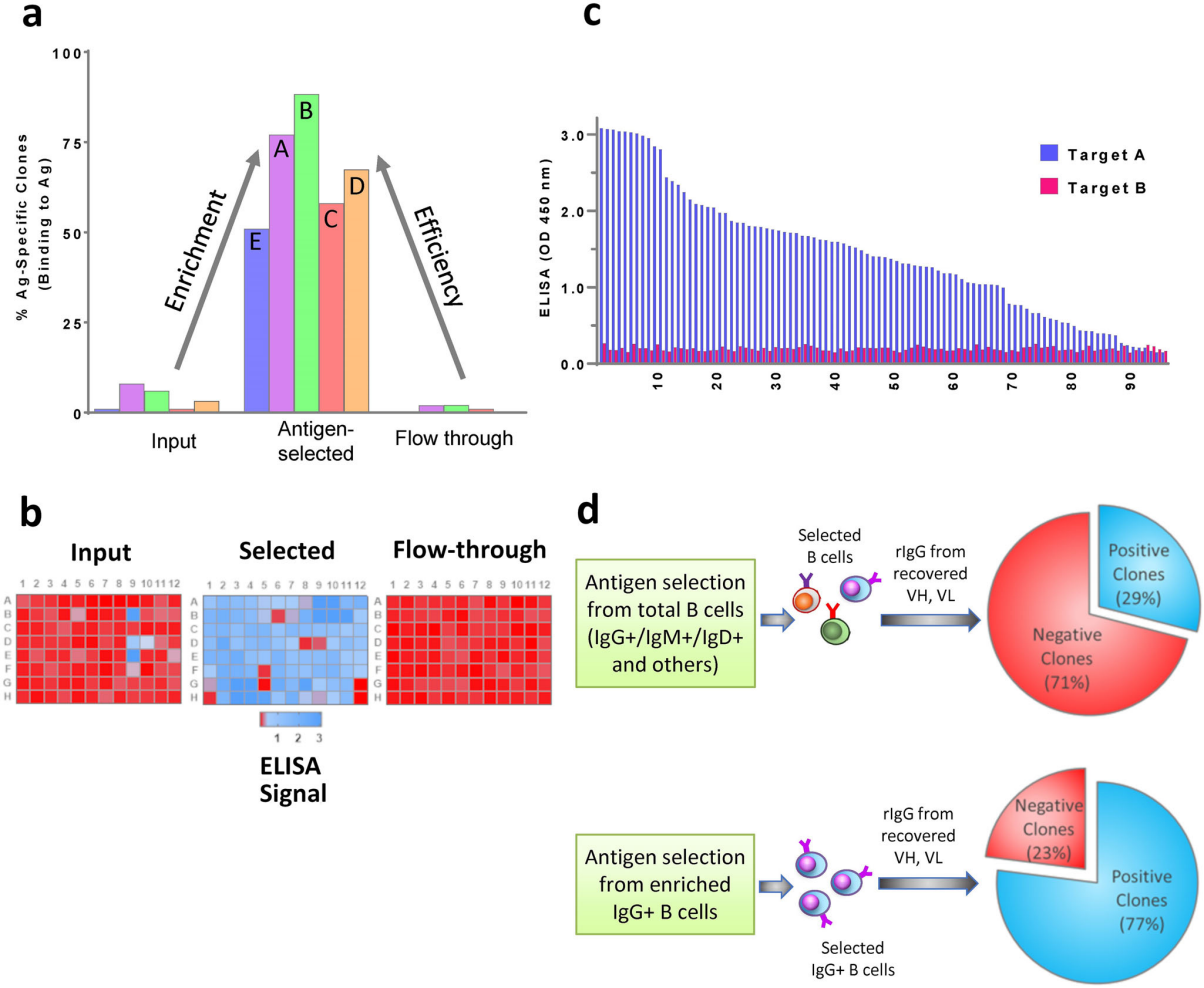
Antigen specificity of rIgG samples from single B-cells obtained before antigen selection (input), after antigen selection and those not selected by the antigen (flow through). **a** ELISA with individual recombinant antibodies expressed in Expi293F cells, demonstrates that target-specific antibodies are highly enriched in antigen-selected B-cell population as compared to the input and flow-through populations. Percentage of samples reacting specifically to the target antigen from five different studies, each using a different target antigen (A, B, C, D and E) are demonstrated in the bar graph. **b** Representative heatmaps show ELISA binding results of individual recombinant antibodies recovered and expressed in 96-well format from input, antigen-selected and flow-through B-cell populations for target A. **c** Individual recombinant antibodies expressed from 92 antigen-specific B cells for target A demonstrate specific reactivity only to target A in ELISA. These antibodies did not show binding to target B which has the same tag as target A. **d** Panel shows depletion of B cells expressing cell-surface IgM and IgD from the input B-cell population increases percentage of antigen positive clones.

Supernatants containing individual antibodies secreted from transfected Expi293F cells were screened for binding to the recombinant target antigens by ELISA. The results indicated that our approach was very effective in enriching target-specific B cells. 51–88% rIgGs from the antigen-selected cells bound to cognate target antigens. Specifically, antigen positive frequency for different antigens are as follows: A (77%), B (88%), C (58%), D (67%) and E (51) (Fig. 3a, b). In contrast, target-specific IgGs in the input and flow-through fractions were 1–8% and 0–2%, respectively, (Fig. 3a, b). The small percentage of target specific IgGs in the flow through population demonstrated that the antigen specific enrichment of B cells worked efficiently and nearly to completion.

We also investigated specificity of the antibodies by screening for reactivity to non-relevant proteins. Figure 3c demonstrated that antibodies isolated from AgSC for target A is highly specific to that antigen and minimally cross-reacted to a different protein with the same tag. Similar experiments were also done for the remaining four targets, antigens B, C, D and E, which showed none to minimal cross-reactivity to a non-relevant protein with the same tag.

### Depletion of IgM and IgD expressing B cells is critical for efficient recovery of antigen-specific IgG recovery

To improve isolation of antigen-specific IgGs we excluded B cells that expressed IgM or IgD on the surface and ensured that a highly enriched IgG^+^ B-cell population was selected. We depleted B cells expressing IgM and IgD on the surface by using biotinylated anti-mouse IgM and anti-mouse IgD antibodies, which bind specifically with the heavy chains of mouse IgM and IgD, respectively. After magnetic separation of IgM^+^ and IgD^+^ B cells, unlabeled IgG^+^ B cells were collected. Use of total B cells (TBC) as the input for antigen-specific B-cell selection, yielded a recovery rate of paired antibody V genes ≤50%. However, when IgG^+^ B cells (IgM^+^ and IgD^+^ B cells depleted) were used as the input cells, the recovery of paired antibody V-genes increased to ≥90% (Figs. 2c, 3d). This difference was also reflected when percentage of antigen positive clones were calculated. Antigen-specific selection (for target A) from input B cells without prior depletion of IgM^+^ and IgD^+^ cells resulted in a low percentage of antigen specific clones (29%). Whereas, when enriched IgG^+^ B cells (with prior depletion of IgM^+^ and IgD^+^ cells) were used as input for antigen selection, there was a significant increase in the percentage of antigen specific clones (77%) (Fig. 3d). These results indicate that prior depletion of IgM^+^ and IgD^+^ B cells significantly improves antigen specific B-cell selection process and enhances subsequent recovery and identification of target-specific clones.

### Immunized phage libraries

Current plate-based B-cell cloning and antibody screening technologies do not allow for phenotypic assay of hundreds of thousands to millions of B cells. To increase the screening power, we generated phage display libraries from IgG^+^ TBCs and AgSCs. We took aliquots of the IgG^+^ TBC and AgSC pools for antigens C and D and made Fab based phage display libraries. We chose antigens C and D for library preparation to test our hypothesis on antigens that differ substantially in their site of expression, wherein, antigen C is expressed as the ectodomain of a cell surface receptor and antigen D is expressed as a soluble antigen. The size of each library was about 10^8^. We then carried out a comparative study of the two libraries for each antigen.

### Unpanned library from AgSCs had higher frequency of binders than TBCs derived library

**Fig. 4:**
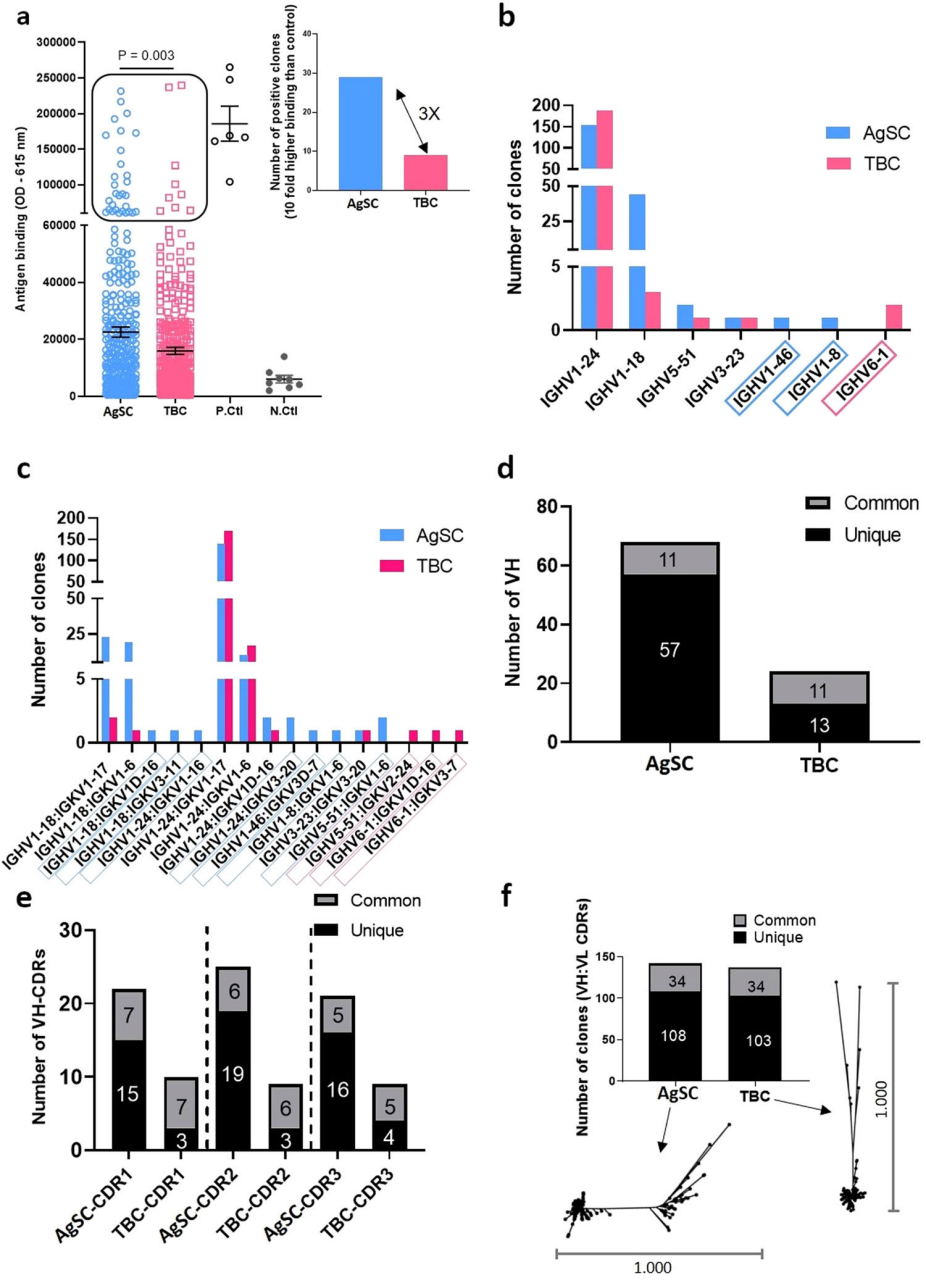
Comparative analysis of antibody clones from AgSC and TBC libraries against antigen-C. **a** Phage ELISA depicts binding to target antigen. Binding by phage particles rescued from individual colonies (*N* = 352 from each library) is depicted as open circles. Positive control (P. Ctl) is M13 phage with target-specific Fab expressed on surface. Negative control (N. Ctl) is M13 phage with anti-trinitrophenal (TNP) Fab expressed on surface. Mean values are represented as horizontal bars and error bars indicate standard error of mean. Statistical analysis is done by two-tailed unpaired t-test. Clones within the black box demonstrate ten-fold higher binding than negative control and the inset shows the number of clones from AgSC (*N* = 29) and TBC (*N* = 9) libraries that are above the set threshold. **b** Graph shows heavy chain V-gene usage by antigen reactive unique clones from AgSC and TBC libraries. Annotation is based on IgBLAST classification. Blue boxes represent V_H_-genes exclusive to clones from AgSC library and pink represents those exclusive to clones from TBC library. **c** V_H_ and V_K_ pairing combinations in clones from the two libraries are presented in bar graph. Blue boxes represent V_H_–V_K_ combinations exclusive to clones from AgSC library and pink represents those exclusive to clones from TBC library. **d** Chart shows number of unique (black) and shared (gray) VH sequences among AgSC and TBC clones, considering all CDRs and frameworks 2–3. **e** Number of unique (black) and shared (gray) CDRs among clones from AgSC and TBC libraries is presented for each of the three V_H_ CDRs. **f** Number of unique (black) and shared (gray) paratopes (all CDR sequences from V_H_ and V_K_ domains) of target-specific antibodies from the two libraries is represented in bar graph. Radial phylogenetic trees showing the diversity among antibody clones from AgSC, and TBC libraries are depicted. The scale represents Juke–Cantor distance model as explained in the methods section. N individual clones.

We first evaluated the specificity of 352 randomly picked clones from each of the two libraries against antigen C by phage ELISA prior to panning and observed that clones from AgSC library displayed significantly higher binding as compared to clones from TBC library (Fig. 4a). The difference was also reflected in the number of clones showing ≥ 10-fold binding over background from AgSC library versus TBC library (Fig. 4a-Inset). In comparison to clones from TBC library, number of clones from AgSC library above the set threshold was three times higher (9 vs 29, respectively).

### AgSC library clones displayed higher number of V_H_-gene usage and unique V_H_/V_K_ pairs

The AgSC and TBC libraries were panned on antigen-coated streptavidin beads for two rounds. The eluates were then used to infect *E.coli*, which were then plated onto agar for isolating single colonies. Each bacterial colony was transferred to an individual well of 96-well plates and infected with M13KO7 helper phage to rescue recombinant phage particles to perform phage-ELISA. Clones were selected based on binding to the antigen and Sanger sequencing was performed to identify unique binders. An Ig-BLAST-based annotation of antibody sequences was performed on 399 unique clones (203 AgSC and 196 TBC clones) that were specific to antigen C. In addition to dominant usage of V_H_-gene family *IGHV1-24* by clones from both the libraries, AgSC library clones also substantially utilized V_H_-gene family *IGHV1-18* (Fig. 4b). Interestingly, two V_H_-genes, *IGHV1-46* and *IGHV1-8*, were found only among AgSC library clones. There was one V_H_-gene, *IGHV6-1* that was found only among TBC clones. We did not observe major difference in number of clones using the dominant V_K_-genes; however, three V_K_-genes, *IGKV1-16*, *IGKV3-11*, and *IGKV3D-7*, were found only among AgSC library clones as compared to two, *IGKV2-24*, and *IGKV3-7*, found only among the TBC library clones. We further evaluated V-gene usage in V_H_/V_K_ pairing (Fig. 4c). Both AgSC and TBC library clones displayed pairing between the prominent V-genes, *IGHV1-24* and *IGKV1-17*, respectively. However, AgSC library clones displayed multiple combinations attributed to the V_H_-gene *IGHV1-18*, which was negligible among TBC library clones. Additionally, multiple unique combinations of V_H_/V_K_ were observed among AgSC library clones as compared to TBC library clones (7 vs 3, blue and pink boxes, respectively in Fig. 4c).

We observed similar results for 127 unique clones (71 AgSC and 56 TBC library clones) that were specific for antigen D (Supplementary Fig. 1). Apart from the dominant usage of V_H_-gene family *IGHV1-18* by clones from both the libraries, substantial number of clones from AgSC library also used V_H_-gene family *IGHV3-11* in comparison to clones from TBC library (17 vs 3, Supplementary Fig. 1a). The usage of *IGHV3-11* gene family also translated into higher number of unique combinations of V_H_/V_K_ among AgSC library clones compared to the TBC library derived clones (9 vs 3, Supplementary Fig. 1b). Interestingly, the gene family *IGHV4-34* that is unique to clones from TBC library and represented in significant numbers resulted in only one pairing combination with *IGKV1-6* (Supplementary Fig. 1b). Similar to antigen C, we did not observe major difference in number of clones using the dominant V_K_-genes for antigen D-specific antibodies; however, one unique V_K_-gene *IGKV2D-29* was used only by clones from AgSC library.

### Next-generation sequencing of V_H_ genes from panning outputs identified similar dominant V_H_ gene families observed in Sanger sequencing along with several new gene families

Phagemids isolated from bacteria infected with phage panning outputs of the AgSC and TBC libraries against antigen D were used to amplify V_H_ gene fragments for NGS. We obtained >50,000 paired reads per pool and the sequence analysis was performed using our internal analysis tool. For our analysis, we only used the sequences that were observed at least twice in the sequencing run. We identified a total of 2000 unique V_H_ sequences in the AgSC library and 2033 unique V_H_ sequences in the TBC library pools. Although, antigen-specificity of these clones were not verified, the dominant gene families observed in the Sanger data (*IGHV1-18*, *IGHV1-24*, *IGHV1-46*, *IGHV3-11*, *IGHV3-33*, and *IGHV4-34*) were similarly represented in the NGS data (Supplementary Fig. 2). Similar to the Sanger data, we observed a high representation of *IGHV3-11* gene family in the NGS data among the AgSC samples (206) in comparison to the TBC clones (121). On the contrary, the gene families *IGHV4-34* and *IGHV6-1*, which were not represented among the AgSC clones in the Sanger data, were observed in the NGS dataset. Additionally, several new gene families (*IGHV1-2*, *IGHV1-8*, *IGHV3-23*, *IGHV3-30*, *IGHV3-48*, *IGHV3-7*, *IGHV4-39*, *IGHV4-59*, *IGHV5-51*) that were not observed in the Sanger data among both the libraries, were observed at a minor level in the NGS dataset (Supplementary Fig. 2). Since the Sanger sequencing was done with antigen specific clones while the NGS was done with the total eluate from panning rounds, it is possible that these sequences represent non-specific background phage. It is also possible that they are low frequency sequences not represented among the samples investigated for antigen specific screening and Sanger sequencing.

### AgSC library clones displayed higher number of unique V_H_ sequences and greater CDR diversity

Using an in-house bioinformatics tool made by excel macros for finding unique sequences and applying IMGT annotations, we first identified unique V_H_ sequences, taking into consideration all CDRs and frameworks 2 and 3. We did not consider frameworks 1 and 4 into our analysis due to the use of degenerate primers, which anneal to these regions for V-gene amplification. Since the source of the two libraries is the same, we also examined antibody V_H_ sequences that are common among the clones from AgSC and TBC libraries. A total of 57 unique V_H_ sequences were found among AgSC library clones as compared to 13 among TBC library clones and 11 were common among both (Fig. 4d). This difference was also reflected when individual CDRs were evaluated. As shown in Fig. 4e the number of unique sequences were 15 vs 3 for CDRH1, 19 vs 3 for CDRH2 and 16 vs 4 for CDRH3 in AgSC and TBC libraries, respectively. Thus, across the different HCDRs, AgSC library derived clones had 4 to 6-fold greater diversity than TBC library derived clones. Similar analysis was performed for V_K_ sequences; however, we did not observe substantial difference in the number of unique V_K_ sequences between clones from AgSC and TBC libraries (147 vs 115), indicating that the immune response against target C was probably largely heavy chain driven. Also, there are a greater number of shared CDRs than unique ones among V_K_ sequences from both libraries. We further evaluated the diversity of unique clones based on all the six CDRs from heavy and light chains together and showed the sequence divergence among the clones using a radial phylogenetic tree (Fig. 4f). Clones from AgSC library formed multiple independent clusters, indicating that they originated from multiple founder clones and further diverged into distant clonalities. In contrast, clones from TBC library mainly formed a single cluster and few diverged offshoot clones. Overall, this indicated that the potential paratopes of antibodies from AgSC library were more diverse than those from TBC library.

### Antigen-based bulk-selection of B cells result in enrichment of biologically functional clones

**Fig. 5:**
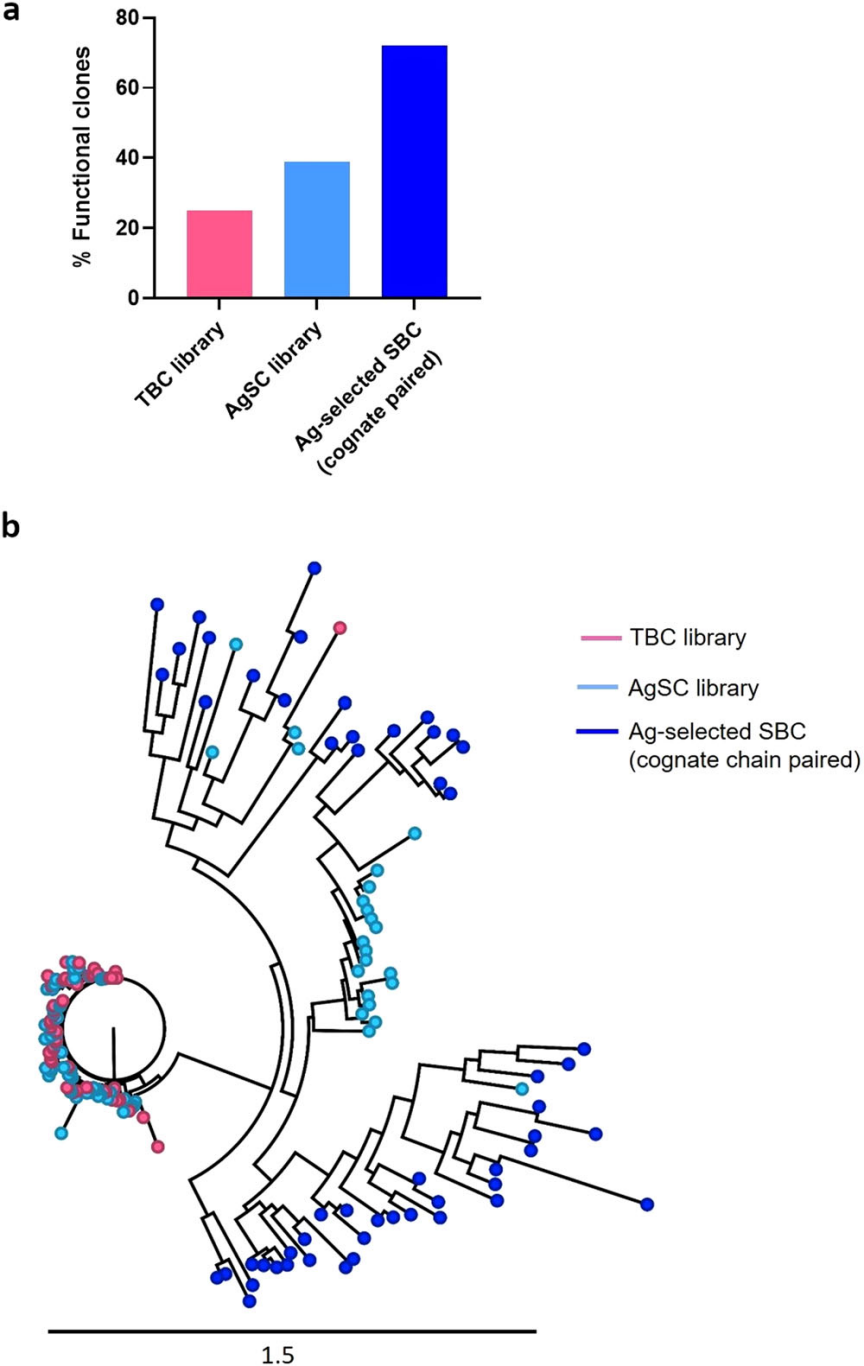
Phylogenetic analysis and mapping of functional antibody clones against antigen C obtained from (i) single B-cells after bulk selection with antigen (cognate chain paired), (ii) AgSC and (iii) TBC libraries. **a** Percent functional antibodies were identified from TBC (49 of total 196 clones or 25%) and AgSC (80 of total 203 clones or 39%) libraries and cognate paired Ag-selected single B cells (54 of total 75 clones or 72%). **b** Radial phylogenetic mapping of functional clones derived from AgSC and TBC libraries, and cognate paired Ag-selected single B cells (SBC) illustrates the diversity of clones from these platforms. The scale represents Juke–Cantor distance model as explained in the methods section. Cognate chain paired clones showed the greatest diversity followed by clones from AgSC and then by clones from TBC libraries. Sequences of clones from AgSC library were more closely related to those with cognate chain paired clones. There was very little overlap between TBC library derived clones with cognate chain paired clones.

Encouraged by the observations that pre-selection of antigen-specific cells yielded significantly greater percentage of positive hits in single B-cell cloning and also in immunized phage library as compared to total IgG^+^ B-cells, we further evaluated whether enrichment of AgSCs translates into enrichment of functional clones (Fig. 5a). For this, monoclonal antibodies against antigen C, which were derived from the three sources, antigen-selected SBC (cognate chain paired), TBC library, and AgSC library, were subjected to an inhibition of receptor-ligand functional assay, wherein, antibody function was measured by their ability to inhibit the production of cytokine by primary immune cells exposed to the ligand expressed on CHO cells. We observed a higher percentage (72%) of functional antibodies from antigen-selected single B-cell cloning where the cognate chain pairing of HC and LC are maintained, and phage clones derived from AgSC library (39%) where combinatorial pairing of HC and LC is restricted to antigen specific cells. In comparison to these, only 25% of clones derived from TBC library were observed to be functional, wherein, combinatorial pairing of HC and LC occurs between both antigen-specific and nonspecific clones. A phylogenetic analysis of all functional antibodies derived from the three sources showed that TBC library was the least diverse with nearly all clones clustered together; AgSC library derived clones were comparatively more diverse, with many clones clustered close to the TBC library derived clones but some were quite distant; and the antigen selected SBC clones with the cognate pairing of chains were the most diverse. (Fig. 5b).

In a second example, antigen D, functional assay was performed using an InvivoGen HEK-Blue cell-line to measure receptor antagonism by colorimetric measurement in a secreted embryonic alkaline phosphatase assay (SEAP) with the InvivoGen QUANTI-Blue detection reagent. In this example we observed significantly higher percentage of functional antibodies against antigen D from the AgSC library in comparison to TBC library (38 vs 14%, Supplementary Fig. 1c). For antigen D, we do not have data from antigen selected SBC from the same mice used for building the phage library. However, when we compared to SBC data obtained from immunized mice of the same cohort, we found 48% of the clones to be neutralizers (Supplementary Fig. 1c). Like in the example of antigen C, we again observed highest percentage of neutralizers coming from the cognate paired clones, followed by immunized library made with AgSCs and the least from library made with TBC. (Supplementary Fig. 1c). The phylogenetic analysis of clones from the three sources indicated greater divergence among clones from SBC, in comparison to those from the libraries (Supplementary Fig. 1d). These results together indicate that pre-enrichment of antigen-specific B cells leads to enrichment of functional clones along with maintaining high diversity. These results indicate that preserving the cognate chain pairing is important for maintaining the functional properties of the antigen-specific antibodies.

### Characterization of recombinant Fab molecules of the functional clones from AgSC and TBC libraries against antigens C and D display differences in expression levels but not in other biophysical properties like thermal stability and affinity

We further characterized the functional clones derived from the two libraries against antigens C and D by producing recombinant Fabs of these clones. We used our Protein Expression and Purification Platform (PEPP) to produce the Fabs and performed expression analysis, thermal stability assay and affinity measurements on these clones. For antigen C, 41 functional clones from AgSC library and 28 functional clones from TBC library were selected for production in high quantities through the PEPP system based on higher diversity in the CDR regions, functional assay performance and absence of sequence liabilities. For antigen D, we did not further triage the functional clones due to lower numbers and used all 27 functional clones from AgSC library and all 8 functional clones from TBC library for production as recombinant Fabs.

Expression data derived from the two libraries displays significantly higher expression levels of recombinant Fabs of the functional clones from AgSC library than from TBC library against antigen C (Supplementary Fig. 3a). Although not statistically significant but a similar trend was observed for functional clones derived from the two libraries against antigen D (Supplementary Fig. 3b). It is to be noted that all recombinant Fabs were derived by transient transfection of expression vectors under identical conditions (see methods).

Since thermal stability of antibodies can be indicative of their solubility and aggregation propensity, we measured the melting temperatures (T_M_) of recombinant Fabs using Nano Differential Scanning Fluorimetry (NanoDSF). We were able to measure the T_M_ of 39 Fabs from AgSC and 26 Fabs from TBC library against antigen C. Likewise, for antigen D we were able to measure the T_M_ for 23 Fabs from AgSC and 7 Fabs from TBC library. For both the antigens, the Fabs from AgSC and TBC libraries were stable as indicated by T_M_ > 50 °C for all clones (Supplementary Fig. 4). We did not observe a significant difference in the thermal stability of Fabs from clones of either libraries against the antigen C or D (Supplementary Fig. 4a, b).

The affinity of recombinant Fabs was measured to identify the difference in binding among clones coming from the two libraries for antigens C and D. We were able to measure the affinity of 40 Fabs from the AgSC library and 28 Fabs from the TBC library against antigen C. Although we did observe five Fabs with higher affinity against antigen C and a higher median affinity among the clones from AgSC library, the difference was not statistically significant in comparison to affinity measurement of Fabs from the TBC library (Supplementary Fig. 5a). For antigen D, we were able to obtain measurable affinity data for 12 Fabs from the AgSC library and 6 Fabs from the TBC library with no statistical difference between the clones from the two libraries (Supplementary Fig. 5b).

## Discussion

The overarching goal of this study was to create a platform agnostic, process improvement for antibody discovery, from immunized sources, by honing on the enrichment of extremely small number of the antigen-specific B cells in a sea of irrelevant B-cells. The study hinges on tapping into the memory B-cell (MBC) repertoire which is the population from which the plasma cell (PC) repertoire is derived^30^^,^^31^. Although we used spleen and lymph nodes of immunized mice for evaluating the concept and highlighting its values, it is understandable that the approach can be applied to memory B-cells of other species. We present results showing that beginning with enrichment of antigen specific MBC it is possible to quickly and effectively isolate diverse functional antibodies by single B-cell cloning and through phage display based combinatorial libraries.

The immune repertoire has billions of B cells. A miniscule fraction of these cells is antigen-specific, most of which are memory B cells expressing IgG on the surface^13–15,32^. This exceedingly tiny fraction of antigen-specific B cells within a huge population of total B cells is akin to a needle in a haystack. Therefore, antibody discovery platforms from immunized animals or convalescent patients carry an enormous baggage of unnecessary screening burden and in fact, do not permit fishing the entire antigen-specific repertoire. Mining of large repertoires even with high throughput screening processes can be technically challenging and cost prohibitive. Bulk selection of antigen-specific B cells mitigates this challenge because it debulks the non-specific clones^24^ and enables screening of the minor population of antigen specific cells, thereby enhancing the probability of better mining the immune repertoire and finding the rare functional antibodies^33^^,^^34^. While flow cytometry using fluorescently tagged antigen is a commonly used technique to isolate antigen-specific B cells, it is known to lead to loss and damage of cells when employed at very high speed^22,23^, which is essential to sort through a very large number of cells. Therefore, an alternate method that can rapidly and gently isolate antigen-specific B cells from secondary lymphoid organs of immunized animals is essential and as discussed below can cater to different discovery platforms such as immunized display libraries, hybridoma and microfluidics-based methods for generating monoclonal antibodies.

Memory B cells (MBC) express membrane anchored immunoglobulins and harbors inside them the genes for the displayed antibody. This is akin to the linkage between phenotype and genotype of display systems and is amenable to phenotypic selection. We used the principle of bio-panning to develop a method wherein biotinylated antigen is used in conjunction with MACS technology to purify antigen-specific MBCs in bulk. Starting from harvested lymphoid organs, the whole procedure takes about 1 hour to purify all antigen-specific MBCs from spleens and lymph nodes of three immunized mice. There are four main steps that involve RBC lysis, removal of T-cells, removal of IgM and IgD expressing B cells leaving only IgG and IgA expressing cells and finally a step in which antigen-specific cells are purified from isotype switched B-cells. Antigen-specific plasma cells are not selected and become part of the ‘flow-through’ population. The percentage of plasma cells (PC) are extremely low and since we observed a small percentage (0–2%) of antigen specific recombinant IgG (r-IgG) from the flow-through population, we interpret that PC do not create a significant loss of antigen-specific pool. We are planning to reduce the time to complete the process further by combining the steps for removal of T-cells and platelets with the step for removal of IgM and IgD cells. In studies by Hermans et al.^34^, and Lamb et al.^24^, the authors describe similar methods of enriching antigen-specific B cells by magnetic separation. In a study by Kivi et al.^33^, antigen specific B-cells were isolated on surface coated with the antigen and in a paper by Chang et al.^35^ adherent cells were used to isolate antigen specific B-cells. Our method reports several key distinctive features. (i) we tested the method on 5 different therapeutic targets as opposed to one target or cell; (ii) report on the characteristics of a variety of recombinant monoclonal antibodies from the enriched cells including their functional properties; (iii) report much higher levels of enrichment up to 51–88% (as opposed to about 40% reported by Lamb et al.), possibly due to the removal of IgM and IgD cells. (iv) in our study we have performed a detailed analysis of the V-gene diversity of antigen-specific B cells and (v) we couple the technology to display libraries and demonstrate the advantage of the method by showing that immune libraries made with AgSC quickly yields a more diverse panel of functional clones than those that are obtained from TBC.

Spleens and lymph nodes from three mice typically yield approximately 3 × 10^8^ cells (Table 1). At the highest achievable cytometry speed of 40,000 cells/sec, isolating antigen-specific B cells from this sample will take about 125 min as compared to 60 min by the MACS method. One can also first purify B cells by MACS and then subject to cytometry-based isolation of antigen-specific MBCs in pool. The calculated time for this procedure will be ~65 min, almost same as the procedure we described. However, both, in our experience as well as reported by others, the loss of cells is substantially more in cytometry than in MACS^23^.

We have validated our method of AgSC purification from groups of human immunoglobulin transgenic mice, immunized separately with different antigens–A, B, C, D and E. In each case, the percentage of positive hits in the starting input population, the antigen selected pool and the flow-through fraction were assessed by generating recombinant IgGs from immunoglobulin expression cassettes^21^ from a panel of single cells in 96-well microtiter plates. As shown in Fig. 3a, b, for each of the target’s A-E, the frequency of antigen-specific r-IgG in the input, the antigen selected and the unselected flow-through populations were between 1 and 8%, 51–88% and 0–2%, respectively. It is noteworthy that the difference in frequency of the selected population compared to the starting and flow-through populations is over a magnitude higher. This indicates the importance of removal of the irrelevant cells prior to screening. As shown in Table 1, only 0.7–3.2% of the IgG^+^ B cells were selected by individual antigens and ~95% or more of the starting cells remain in flow-through. A small fraction of the cells may remain trapped in the tubes and column since the sum of antigen selected and flow through cells is slightly less than the input number of cells. It is also likely that dying cells bind nonspecifically to the tubing and column material and are unaccounted for. The fraction of AgSCs is smaller (0.2–1.1%) when the total B cells were considered. This indicates that in the absence of antigen pre-selection the antibody discovery process is hugely cumbersome and flawed. Pre-selection of the exceedingly low number of AgSCs allows for screening to be focused only on the relevant clones.

Our approach for selection of AgSCs was carefully designed to make it an effective and adoptable technique. Before selecting the AgSCs we removed polyspecific IgM^+^ cells, which may lead to high background. Next, we used recombinant purified antigens, which are site-specifically biotinylated with minimum change, if any, in conformation or function^4^. Since most recombinant antigens have purification tags, we used an excess of a non-relevant antigen with the same tag to deselect tag-specific B cells. Importantly, the in-solution selection eliminated limitations due to steric hindrance, partial denaturation and loss of functional conformation that can result from antigen immobilization on plastic surfaces.

A limitation of this method is that it can only select MBCs which have surface IgG and not plasma cells (PC) which are believed to be more matured and naturally selected product of a humoral response but lack surface IgGs. The results in Fig. 3a, b as well as Table 1 indicate that for each of the five antigens studied there were barely any antigen-specific clones (<2%) in the flow-through population, which should have the plasma cells, if there were any. However, we have not characterized the flow-through cells using surface markers and it might be interesting to know if this fraction really has any plasma cells. We envisage that if there are any plasma cells in spleen or lymph node or if bone marrow is also used as a source of antigen specific clones, then PCs could be captured using the method for forming antibody capture complex on PCs for MACS based purification^36^. Our results show some variation in the percentage enrichment of our antigen specific B-cell selection. This disparity is probably attributed to the quality of the biotinylated targets and the expression and yield of individual IgGs from the Expi293F cells.

There are currently three widely used platforms of antibody discovery from immunized source, each with several variations, and a fourth platform emerging. The first three platforms are hybridoma, single B-cell cloning and immunized phage display library and the fourth is microfluidic based B-cell cloning. For each of these platforms, the bulk selection of AgSCs at the beginning can be of great advantage. In this work we demonstrated the results of a comparative study of the output clones generated from immunized phage library from IgG^+^ AgSCs and TBCs, the latter being a routine choice of immunized phage-display based approaches^17,18^. Immunized display libraries are a preferred method of isolating antigen-specific clones because unlike naïve libraries they do not need to be big in size and can be customized for the antigen of interest. Furthermore, they are quick to make and often yield clones with desired specificities^16–18^. We reasoned that if IgG positive cells from immunized animals have <1% AgSCs^13–15^, then in a combinatorial library made out of IgG^+^ TBCs, any one or both of the following scenarios can happen: (i) antigen-specific cognate chain pairs have a higher risk of being lost; (ii) many artificial clones will be created by pairing of IgG chains from AgSCs with chains from irrelevant cells. Conversely, if libraries are made from AgSCs, then the chances of paired cognate chains have a higher likelihood of being preserved and combinatorial pairing among chains from these cells will create several artificial clones, albeit with higher likelihood of being antigen positive (since they all originate from AgSCs). We based our analysis between the AgSC and TBC libraries using the following criteria: (i) richness of antigen-specific clones in each library and (ii) their clonotype diversity based on germline gene usages, CDR diversity and clusters of paratopes as predicted by unique combination of all 6 CDRs.

We generated AgSC and TBC libraries for two target antigens (C and D) and tested the enriched clones for various parameters like, binding, function and V-gene diversity. For both the antigens, AgSC-library derived clones were much superior in all the above parameters. For simplicity of discussion and because of the similarity in our observation for both antigens, we largely focus on discussing the results from clones against antigen C and briefly discussed about the clones against antigen D. Distinctive and advantageous features of AgSC library versus TBC library against antigen C were observed in our comparison (Fig. 4). Even prior to panning, AgSC library was found to be not only more enriched in antigen-specific clones than TBC library but also had clones that demonstrated higher binding (Fig. 4a). However, like any phage ELISA, strength of binding can be influenced by expression/display level on the phage surface, and we have not determined this on the hundreds of clones that we analyzed. In terms of clonotypes, as defined by V_H_ sequences, we demonstrated that both AgSC and TBC library-derived clones had abundant representatives of the germline *IGHV1-24* (Fig. 4b), which could be a result of sequestration of the immune response towards few gene families. Since the source of the two libraries was the same, emergence of similar dominant families was expected. Surprisingly, the 2nd most dominant V_H_ germline *IGHV1-18* had a disproportionately higher representation among AgSC clones as compared to TBC clones. In addition, rare V_H_ germline genes *IGHV1-46* and *IGHV1-8* were exclusively represented among AgSC clones, possibly due to loss in TBC library through non-productive pairing with irrelevant light chains coming from cells not specific to the antigen. We think that the reason for low or no representation of certain VH germlines among TBC clones could be due to unproductive pairing of these heavy chains with light chains from irrelevant cells (not antigen specific) in the TBC pool. However, TBC clones did have one unique germline, namely *IGHV6-1*, that was absent among AgSC clones. It is possible that we did not observe *IGHV6-1* in AgSC library because it probably did not exist among AgSCs but was derived by pairing of an originally irrelevant (not antigen specific) HC with an antigen-specific LC from AgSC.

The difference between the two libraries becomes even more prominent if clonotypes are defined by V_H_/V_K_ pairing (Fig. 4c). Understandably, being the dominant V_H_ and V_K_ genes, *IGHV1-24/IGKV1-17* combination was the most prevalent among clones from both libraries. However, AgSC library also had a significant number of clones with different V_H_/V_K_ combinations. In fact, this antigen-specific library had 7 unique combinations of V_H_/V_K_ compared to 3 from the TBC library. Indeed, the considerable difference in number of unique V_H_/V_K_ pairing between AgSC and TBC library derived clones is directly attributed to the high representation of V_H_ germline gene family *IGHV1-18* that combines with various LCs, and to the presence of rare V_H_ genes *IGHV1-46* and *IGHV1-8* in AgSC library. The availability of several V_H_/V_K_ combinations among AgSC clones could be due to productive pairing among the V_H_ and V_K_ chains of antigen specific clones. In contrast, it is likely that combinatorial pairing of antigen-specific V_H_/V_K_ with chains from irrelevant cells could have resulted in loss of productive V_H_–V_K_ combinations.

In terms of distinct sequences, we again observed greater uniqueness in clones from the AgSC library, which displayed greater V_H_ diversity than those derived from TBC library (Fig. 4c). We also observed that most of the mutations in the unique antibodies were restricted to CDRs rather than frameworks and this distinction was higher in AgSC library derived clones. Heavy chain CDR3s have been repeatedly reported to be the most important region in antibody-antigen interaction and govern the affinity^37,38^. Our comparison demonstrates greater diversity in HCDR3 among AgSC clones than TBC clones (16 vs 4), which ultimately contributes to the higher diversity of AgSC clones. We did not see such dramatic differences in V_K_ sequences in clones from the two different libraries, which indicates that the immune response in mice against antigen C was probably largely governed by introduction of mutations in heavy chains. Finally, we wanted to see if the resulting differences in mutation frequency influences the overall diversity of clones from AgSC and TBC libraries. As shown in Fig. 4f, when unique clones from the AgSC and TBC libraries were segregated according to mutations in their paratopes (groups of clones with identical CDRs) we observed that the number of groups are very similar between the 2 libraries, 108 for AgSC library and 103 for TBC library. However, when we studied the phylogenetic relationships among clones from each library, we found that the diversity observed in clones from AgSC library constitutes an array of founder clones, in contrast to the TBC library which harbors very limited founder clones. Together these results suggest that library generated from AgSCs results in higher diversity of clones and clonotypes compared to a library generated using TBC.

We also performed deep sequencing on the bacterial clone pools from panning rounds of the AgSC and TBC library against antigen D, which provided an in-depth information on the V_H_ gene repertoire. The NGS data re-affirmed several observations of Sanger sequencing and provided new insights too. We identified 2000 unique clones from the AgSC library and 2033 unique clones from the TBC library. Although antigen-specificity of these VH clones is not known, they provide an understanding of the preferential gene-usage against antigen D. We observed that the dominant V_H_ gene families observed in the Sanger data were also highly represented in the NGS data (Supplementary Fig. 1 and Supplementary Fig. 2). Interestingly, the V_H_ gene family *IGHV3-11*, which was observed in the Sanger’s data to be used at a higher rate by the AgSC library clones and made several unique pairs with multiple light chains (Supplementary Fig. 1), was also represented at a comparatively high rate in the NGS data (Supplementary Fig. 2). Contrary to the Sanger data, we observed that one of the major gene family *IGHV4-34*, which was exclusively observed among TBC library clones was equally represented among the AgSC library clones in the NGS data. We postulate that this difference between the Sanger and NGS data could be a result of either under-sampling of clones during selection or due to a gain of functionality by the TBC clones by productive pairing with other light chains. We do, however, understand the limitation of our assumption because unlike the Sanger data, NGS data did not provide cognate-chain pairing information due to the limitation of Illumina sequencing read lengths. Additionally, we identified minor representation of nine V_H_ gene families in the NGS dataset. NGS-based deep repertoire mining could potentially help identify higher number of antigen-specific clones with greater diversity, and long-read sequencing techniques like Pac-Bio could be helpful in identifying cognately paired antibody.

We further investigated whether enrichment of antigen-specific B cells also results in greater proportion of antibodies that are efficient in mediating a biological function. Indeed, antibodies selected against antigens C and D from antigen-specific single B-cells and AgSC library consist of higher percentage of clones that are functional in comparison to clones from TBC library (Fig. 5a, Supplementary Fig. 1c). When we further compared the frequency of functional clones from combinatorially paired antibodies versus the cognate paired antibodies, we observed higher percentage of functional clones in the latter. We propose three main reasons for the above observations; (1) pre-enrichment of AgSCs allows deeper screening of relevant clones and results in higher availability of functional clones from a smaller but relevant pool of cells; (2) even if cognate chain pairing is lost, combinatorial pairing of chains among AgSCs results in significant improvement in binding, sequence, paratope and functional diversity as opposed to combinatorial pairing of chains between antigen specific and irrelevant cells in TBC and (3) cognate chain pairing is the most efficient method to preserve function as evident from our observation of single cell VH-VL cloning after bulk isolation of antigen specific cells. We would, however, argue that the choice between deriving clones from combinatorial pairing in AgSC library and/or cognate paired SBC depends upon the number of antibodies and diversity required. Although the frequency of functional clones from cognate paired chains in single cell cloning after bulk antigen selection is higher; in terms of numbers, the combinatorial pairing in AgSC libraries provided much larger number of functional antibodies. This is probably because of the higher throughputness of phage libraries compared to SBC cloning. We indeed observed that high diversity is a persistent feature among functional clones from both cognate and combinatorially paired antibodies from AgSCs. Together, these findings indicate that antigen-bulk selection of B cells is an efficient method over traditional mAb selection from TBCs, as it ensures high diversity and functionality of antibodies.

We further characterized the functional antibodies from the two libraries for expression, thermal stability, and affinity. Recombinant Fabs from the AgSC library against antigen C displayed significantly higher expression levels than the Fabs from the TBC library (Supplementary Fig. 3a). Although not statistically significant, we did observe high levels of expression of Fabs from the AgSC library against antigen D (Supplementary Fig. 3b). The lower expression levels of the TBC library clones could be due to the expression constrains caused by the combinatorial pairing of the heavy and light chains, however, a thorough investigation is further needed to delineate the biophysical characteristics governing these differences. In terms of thermal stability and affinity, no differences were observed from the Fabs of either library (Supplementary Fig. 4, 5). However, compared to antigen D, the affinities of the Fabs against antigen C were considerably lower. We do not precisely know the underlying reason. We have 2 speculations. (i) Because antigen C had 3 to 4-fold lower immune serum titer and because the frequency of AgSC was relatively low compared to antigen D (Fig. 3a) we speculate that there was more combinatorial pairing in the libraries made for antigen C and this might have affected the affinity. (ii) Affinities were measured only for functional clones, and we do not know if there were non-functional but higher affinity clones in the libraries for antigen C.

We envision that the antigen-specific B-cell bulk selection technique could be easily adaptable to other antibody discovery approaches. One example is the hybridoma technology, which is plagued by the extremely low fusion efficiency^39^, resulting in great loss of specific B cells. While the pre-selection of antigen-specific MBCs will not mitigate the low fusion efficiency, one can envision that the vast majority of the resulting hybridoma clones are likely to be antigen-specific and would not need large-scale screening to identify them. Microfluidic droplet-based natural immune repertoire recovery and display^25–27^ and other chip-based^40,41^ microfluidic methods are becoming popular platforms for repertoire deep mining while maintaining cognate pairing during antibody discovery campaigns. These platforms can handle only about 1–2 million B cells at a time, necessitating not only iterative rounds of encapsulation and screening but also limiting mining to only a fraction of the entire repertoire. Pre-selection of antigen-specific B cells will make these platforms rapid and robust by removing the irrelevant cells and capturing cognately paired antibody genes of only the antigen-specific cells.

The use of antigen-specific pre-selection of B cell will expand the possibility to deep-mine the target-specific antibody repertoire. This approach holds the potential to allow simple and rapid selection and enrichment of highly pure antigen-specific B cells from physiological or pathophysiological samples including immune tissues and blood. Use of this method will be helpful to isolate rare B cells expressing surface antibodies with functional attributes. This selection strategy is powerful–yet flexible to scale up regardless of the source of the B cells. As such, this technique can be easily adopted in most laboratory settings. Automation with high-throughput screening, built around the antigen-specific B-cell pre-selection technique, would accelerate and streamline the antibody discovery process. Integration of automated processes would increase the throughput, enabling broader repertoire coverage and parallel processing of multiple targets, thus reducing overall antibody discovery timeline and cost.

## Materials and methods

### Mice and immunization

About eight to ten-week-old Trianni mice (HHKK) (Trianni, San Francisco, USA)^42^ were used in all studies. All experiments were approved and conducted according to the guidelines of Sanofi Genzyme Institutional Animal Care and Use Committee. Mice were immunized with 50 µg of antigen in adjuvant (Sigma, catalog no. S6322) by intraperitoneal injection, and booster immunizations were performed for four to six times in two-week intervals. Sera were collected after third and last immunization to assess the antibody titers. The mice were rested for three weeks and boosted three days before harvesting secondary lymphoid tissues for processing B cells for antigen selection.

### Biotinylation of target antigen

For site-specific biotinylation in a cellular system, Expi293F cells were co-transfected with Avi-tagged target antigen and biotin ligase (BirA) expression constructs in a total of 200 µg DNA (10:1 ratio of target protein and BirA DNA) in 200 ml culture volume (3 × 10^6^ cells/ml) using ExpiFectamine 293 reagent (Fisher Scientific, catalog no. A14524) (2.7 µl per 1 mg DNA). The media was supplemented with 50 µM (final conc.) biotin. A cocktail of total 10 ml Enhancer solutions (1:10 ratio of Enhancer 1 and Enhancer 2, provided with the ExpiFectamine 293 reagent) was added 24 h post-transfection. Cell-expressed biotinylated protein was purified by standard purification methods and the extent of biotinylation was measured by mass spectrophotometry.

### Preparation of murine immune cells

The mice were sacrificed three days after the final boost and spleens and lymph nodes from three mice were removed and placed in 5 ml of DMEM with 5% FBS. Single cell preparation was made by dissociating cells from the spleens and lymph nodes through a 40 µm nylon strainer (Fisher Scientific, catalog no. 07-201-430). The cells were washed with DMEM, 5% FBS and centrifuged at 500 g for 5 min. The supernatant was aspirated, and RBCs were removed by re-suspending the cells in 2 ml of Hybri-Max RBC lysis solution (Sigma Aldrich, catalog no. R7757) at 37 °C for 2 min. Cells were washed with 30 ml of PBS, 0.5% BSA. Cell count and viability were determined by Vi-Cell XR Cell Counter (Beckman Coulter, catalog no. 731196). Washed cells were centrifuged at 500 g for 5 min and re-suspended in PBS + 0.5% BSA at 1 × 10^8^ cells/ml. These cells were used for B-cell enrichment and subsequent Ag-specific B-cell isolation.

### B-cell isolation

B cells (CD19+, CD19+ CD138+ and CD138+) including plasma cells were isolated by immunomagnetic negative selection from total lymphocyte suspension by using EasySep Mouse B-Cell Isolation Kit (Stem Cell Technologies, catalog no. 19854). In brief, lymphocyte suspension was prepared (1 × 10^8^ cells/ml in 0.25–2 ml PBS, 0.5% BSA) in 5-ml polystyrene round-bottom tube (12 × 75 mm) (Stem Cell Technologies, catalog no. 38007) and rat serum was added (50 µl/ml of cell suspension). Isolation cocktail containing biotinylated antibodies recognizing specific cell surface markers for non-B cells was added to the sample (50 µl/ml of cell sample) and mixed and incubated for 5 min at RT. Magnetic beads (EasySep Streptavidin RapidSpheres 50001) were added to the sample (50 µl/ml of cell suspension), mixed and incubated for 3 min at RT. The volume of the sample was made to 2.5 ml by adding PBS, 0.5% BSA. Non-B cells labeled with biotinylated antibodies and streptavidin-coated magnetic particles were discarded by magnetic separation (EasySep Magnet, Stem Cell Technologies, catalog no. 18000) and total B cells were isolated in solution. Cell number was determined using Vi-cell XR cell counter (Beckman Coulter).

### Enrichment of IgG^+^ B cells by depleting IgM^+^/IgD^+^ B-cell

Mouse B cells expressing surface IgG were isolated from the total B-cell population by depleting B cells expressing surface IgM and IgD. Total B-cell suspension was prepared at a cell concentration of 0.5 × 10^8^ to 1 × 10^8^ cells/ml in 0.5 ml PBS + 0.5% BSA. A cocktail of anti-mouse IgM and anti-mouse IgD antibodies, both attached to streptavidin microbeads were prepared by mixing 20 µl biotinylated anti-mouse IgD monoclonal antibody (clone 11-26c, Fisher Scientific, catalog no. 50-112-8799) with 80 µl of Streptavidin microbeads (Miltenyi Biotec, catalog no. 130-048-101) and then incubated for 30 min at 4 °C. To this solution, 100 µl of anti-mouse IgM micro-beads (Miltenyi Biotec, catalog no. 130-047-301) was added. The IgD/IgM antibody cocktail (100 µl) was added to the total B-cell suspension, mixed gently and incubated for 15 min at room temperature. The cell suspension was then added to a pre-equilibrated (with PBS, 1X, 0.5% BSA) MACS LS column (Miltenyi Biotec, catalog no. 130-042-401) in the magnetic field of a MidiMacs separator unit (Miltenyi Biotec, catalog no. 130-042-302). Flow-through and wash fractions (with 5 ×5 ml of PBS, 1X, 0.5% BSA) from this column were collected as IgG^+^ B cells (unlabeled cells, separated by negative selection) and the cells were counted using Vi-cell-XR cell counter. The column was removed from magnetic field and labelled cells were eluted by flushing out with PBS, 1X, 0.5% BSA from the column. These are IgM^+^/IgD^+^ B-cell population.

### Isolation of antigen-specific B cells

We adopted an in-solution binding and column-based separation method to isolate and enrich AgSC from IgG^+^ B cells. The IgG^+^ B-cell suspension was centrifuged at 500 g for 5 min and re-suspended in PBS, 0.5% BSA at a density of ~0.5 × 10^8^ cells/ml (in 0.25–0.5 ml). At this stage a fraction of these cells (~0.1 × 10^7^) was set aside as unselected “input cells”. The remaining IgG^+^ B cells were used to isolate AgSC. To the IgG^+^ B cells, unlabeled non-relevant antigen (5 µg per 10^7^ cells) was added to capture and remove any non-specific and anti-tag specific B cells (if tagged antigen was used for immunization). The cell suspension was incubated at RT for 30 min with occasional mixing. Then 1 µg of the biotinylated target antigen was added and incubated for 30 min at RT with occasional mixing. Homogenously suspended 100 µl (per 10^7^ cells) streptavidin microbeads (Miltenyi Biotec, catalog no. 130-048-101) were added and incubated for 30 min at room temperature. A MACS LS separation column (Miltenyi Biotec catalog no. 130-042-401) was placed in the magnetic field of a MidiMacs separator unit (Miltenyi Biotec, catalog no. 130-042-302,) and equilibrated with PBS, 0.5% BSA. B cells-antigen-magnetic bead mixture was added onto the LS column. Unlabeled non-specific B cells (“unselected fraction”) were collected as flow-through fraction. The column was washed with 3 × 3 ml PBS, 0.5% BSA and the effluent was also collected as the total flow-through fraction (along with the first column effluent). The column was then removed from the MidiMacs separator and placed on a 15-ml tube. Magnetically labeled-B cells were flushed out of the column by pushing 5 ml of PBS, 0.5% BSA with the plunger into the column. The collected cell population is the antigen-specific bulk B-cell fraction. The number and viability of cells in each fraction was counted using Vi-cell XR counter.

### Single B-cell sorting

Input cells (unselected IgG^+^ B cells before adding antigen), Ag-specific B cells (selected B cells that bound to the antigen) and flow-through (B cells that did not bind to the antigen) fractions were transferred (250 µl cell suspension in PBS, 0.5% BSA) to 5-ml, 12 × 75 mm polypropylene tubes (STEMCELL, catalog no. 38007). The cell fractions were incubated with 3 µl of 7-AAD (7-amino actinomycin D) staining solution (ThermoFisher, catalogue no. 00-6993-50) for 5 min at RT to exclude dead cells during sorting. Single cells were sorted into wells of 96-well plates using BD Influx instrument (BD Biosciences) set with a 70-micron nozzle and 45 PSI pressure.

### Antibody cDNA generation

Individual B cells were placed into each well of 96-well PCR plates containing 6 μl/well of RT reaction buffer that included 1 µl random hexamer (150 ng/µl) (GeneLink, catalog no. 26-4000-03), 0.6 µl 10 mM dNTP (NEB, catalog no. N0447L), 0.14 µl RNaseOUT (40 U/µl) (ThermoFisher, catalog no. 10777019), 0.6 µl DTT (100 mM) (ThermoFisher, catalog no. 50-255-872), 0.5 µl Igepal (5%) (Sigma-Aldrich, catalog no. I8896), 0.012 µl BSA (50 mg/ml) (ThermoFisher, catalog no. AM2616), 1.2 µl 5x RT Buffer, 0.2 µl Super Script III reverse transcriptase (200 U/µl) (ThermoFisher, catalog no. 18080085) and 1.75 µl UltraPure DEPC-Treated Water (ThermoFisher, catalog no. 750024). The RT reaction was carried out with a reaction cycle of 55 °C for 5 min, 25 °C for 10 min and 55 °C for 30 min. The plates were stored at −80 °C until used in the next step.

### Recovery of Ig variable region transcripts from antigen-specific B cells

The genes encoding IgG V_H_ and V_K_ chains were amplified using the cDNA generated from single-sorted B-cell, as the template. The V_H_ and V_K_ genes were amplified separately by two rounds of PCRs in 96-well plates. The first PCR (PCR1) included 2 μl of cDNA, with AmpliTaq Gold 360 Master Mix (ThermoFisher, catalog no. 4398881) 12.5 µl (1X), GC Enhancer 0.5 µl (2%), nested primer pool (5 μM/each) 0.5 µl (0.1 µM) and dH_2_0 up to 25 µl. PCR1 was performed at 95 °C for 5 min (initial denaturation) followed by 35 cycles of 95 °C for 30 sec (cycle denaturation), 55 °C for 30 sec (primer annealing), 72 °C for 1 min (primer extension), and final extension at 72 °C for 7 min. The second round of PCR (PCR2) was performed using 2 μl PCR1 DNA product as the template with AmpliTaq Gold 360 Master Mix 12.5 µl (1X), GC Enhancer 0.5 µl (2%), nested PCR2 primer pool (5 μM/each) 0.5 µl (0.1 µM) and dH_2_0 up to 25 µl. PCR2 was performed at 95 °C for 5 min (initial denaturation) followed by 35 cycles of 95 °C for 30 sec (cycle denaturation), 58 °C for 30 sec (primer annealing), 72 °C for 1 min (primer extension), and final extension at 72 °C for 7 min. The primer set used for PCR2 had overlapping sequences at the 5′ end, which allowed generation of linear expression cassettes^21^ for both V_H_ and V_K_ genes by overlapping PCR. PCR2 products were analyzed for paired V_H_ and V_K_ genes of each IgG by eGel (96-well). The heavy and kappa chain PCR2 products were cleaned up by using HT ExoSAP-IT Fast High-Throughput PCR Product Cleanup reagent (ThermoFisher, catalog no. 785951EA). These DNA products were used for generating V_H_ and V_K_ linear expression cassettes (LEC)^21^.

### Construction of linear expression cassettes (LECs) for IgG

For high throughput expression and binding analysis of natively paired V_H_/V_K_ genes recovered from single B cells, linear expression cassettes (LECs) were generated by overlapping PCR. The transcriptionally active expression cassette was assembled by using three overlapping DNA fragments–the CMV promoter fragment, the V_H_ or V_K_ fragment and the C_H_ or C_K_ region along with the rabbit beta globin polyA signal sequence, as described previously^21^. The CMV fragment and C_H_ and C_k_ polyA fragments were PCR amplified by AccuPrime Pfx DNA polymerase (ThermoFisher, catalog no. 12344024). In brief, in 50 µl reaction 5 μl of 10X AccuPrime reaction buffer, 1 ng template plasmid, 10 pmol of each primer and 1 unit of polymerase were mixed and amplified. The condition for amplification was 95 °C for 3 min initial denaturation, 27 cycles of denaturation at 95 °C for 17 sec, annealing at 61 °C for 30 sec and extension at 68 °C for 80 sec and an additional cycle of final extension at 68 °C for 2 min. The expression cassettes were generated for each pair of H and K genes in 50 µl PCR containing 10 ng each of CMV, V_H_ and C_H_-polyA (for heavy chain) or CMV, V_K_ and C_K_-polyA (for kappa chain), 5 μl 10X PCR buffer, 10 µmol dNTP, 10 pmol of forward and reverse primers and 1 unit of KOD DNA polymerase (Fisher Scientific, catalog no. 710864). The amplification condition was - 95 °C for 2 min initial denaturation, 27 cycles of denaturation at 95 °C for 20 sec, annealing at 62 °C for 12 sec and extension at 70 °C for 70 sec and an additional cycle of final extension at 70 °C for 2 min. The LECs were analyzed by eGel, purified by MinElute 96 UF PCR purification system (Qiagen, catalog no. 28053) and quantified by NanoDrop8000 (ThermoFisher).

### Recombinant antibody generation by Expi293F expression system

IgG heavy and kappa chains of individual antibodies were expressed in Expi293F suspension culture (Fisher Scientific, catalog no. A14527) grown in Expi293F Expression Medium (Fisher Scientific, catalog no. A1435101). Cells were split to a density of 3×10^6^ cells/ml the day before transfection so that the Expi293F cells reached to a density of ~5 × 10^6^ cells/ml, viability >95% on the day of transfection. Cell suspension (0.5 ml) was prepared to a final density of 3 × 10^6^ cells/ml with Fresh Expi293 media into each well of 96-deep well culture plate (Fisher Scientific, catalog no. 07-000-873). Paired IgG1 heavy and kappa chain linear expression cassettes (LECs) were mixed (500 ng each) in 50 µl of Opti-MEM I medium. Separately, 5 µl ExpiFectamine 293 Reagent (Fisher Scientific, catalog no. A14524) was diluted in 50 µl of Opti-MEM (Fisher Scientific, catalog no. 31985062). The diluted ExpiFectamine 293 Reagent was then mixed with the DNA mix and incubated for 20 min at room temperature. This transfection mix was added to the Expi293F cell suspension in each well of the culture plate and mixed gently. The culture plate was sealed with porous Aeraseal film (Sigma Aldrich, catalog no. A9224-50EA) and incubated at 37 °C, 8% CO_2_, 900 rpm in a humidified (≥80%) incubator. After 18 h, a cocktail of Enhancer I and II (comes with the ExpiFectamine 293 Reagent kit; 5 µl of Enhancer 1 and 50 µl of Enhancer 2) was added to each well. The cells were incubated for another 120 h in 37 °C, 8% CO_2_, 900 rpm in a humidified (≥80%) incubator. Cell culture supernatant was harvested by centrifuging the culture plates at 1000 g for 30 min and the supernatants were transferred to Matrix 96-well storage tubes (Fisher Scientific, catalog no. 50-823-846). Antibody concentration in the culture supernatant was measured by Octet (ForteBio) and specific binding to the target antigen was tested by ELISA.

### Screening for antigen-specificity by ELISA

Individual IgGs expressed in Expi293F cells were used to screen for antigen-specific binding by ELISA. Target and control antigens were coated onto Immulon 4HBX ELISA plate (Fisher Scientific, catalog no. 3855) at 5 μg/ml (in PBS), 100 μl/well. The plates were sealed with Nunc Sealers (Fisher Scientific, catalog no. 12-565-71) and incubated at 4 °C overnight. The plate was washed with PBS and blocked with 200 μl/well of blocking buffer (0.5% Probumin Bovine Serum Albumin, Life Science Grade, EMD Millipore, catalog no. 821001) and incubated for 1 hour at 37 °C. To each well of test and control antigen coated plates, 100 μl of individual antibody supernatant (Expi293F sups) was added and incubated at room temperature for 2 h. The plate was washed three times with PBS. To each well 100 μl of anti-mouse IgG (H + L) (Jackson ImmunoResearch catalog no. 115-035-166), diluted 3000X in blocking buffer was added and incubated at room temperature for 1 hour. The plate was washed (3X) with PBS. Turbo TMB HRP detection substrate (Fisher Scientific, catalog no. PI34022) was added to the plate (100 μl/well) and incubated at room temperature for 20 min. The chromogenic reaction was stopped by adding 100 µl (per well) of Stop Reagent for TMB (Fisher Scientific, catalog no. NC0213329). The plate was read at 450 nm using Envision Plate Reader (Perkin Elmer).

### Generation of immune libraries

Phage libraries expressing Fab fragments were generated from IgG^+^ AgSC and TBC isolated from mice immunized with the target antigen C and D. After the isolation of AgSC and TBC, cells were centrifuged and resuspended in RNA lysis buffer to isolate total RNA, using manufacturer’s protocol (Qiagen, catalog no. 74034, 74134). Total RNA was then reverse transcribed to amplify human V_H_ and V_K_ chains using gene specific primers (Table 2) targeting the constant regions, according to the protocol provided in the kit (Invitrogen, catalog no. 18080093). Further, nested PCR was performed in two rounds. In PCR1, cDNA from RT reaction was used in separate reactions to amplify V_H_ and V_K_ genes with forward primers targeting nine V_H_ and eight V_K_ leader sequences that encompass all V-genes in Trianni mice, together with reverse primers used for RT. The primers were designed with degenerate nucleotides to facilitate the amplification of all V_H_ and V_K_ genes. Briefly, in a 25 µl reaction volume, 2.5 µl of cDNA was mixed with 0.5 µl 10 mM dNTPs (Invitrogen, catalog no. 18427088), 0.5 µl each of 10 µM forward and reverse primers, 0.25 µl of Q5 hot-start polymerase (NEB, catalog no. M0493L), 5 µl Q5 reaction buffer and 15.75 µl nuclease-free water; PCR was performed at 98 °C for 30 sec (initial denaturation) followed by 30 cycles of 98 °C for 10 sec (cycle denaturation), 72 °C (V_H_) and 68 °C (V_K_) for 30 sec (primer annealing), 72 °C for 30 sec (primer extension), and final extension at 72 °C for 5 min. A PCR2 was performed with degenerate primers targeting FR1 and FR4 of V_H_ and V_K_ genes consisting of restriction site for cloning into phagemid. All the reagents for PCR2 were doubled than PCR1 to accommodate a 50 µl reaction volume to obtain enough DNA for cloning. Template DNA (1 µl each) from PCR1 were added to each tube in a multiplex format. After PCR2, variable region gene fragments were gel purified (Macherey–Nagel, catalog no. 740609) followed by double-digestion of V_H_ fragments with restriction enzymes SspDI (Thermo Scientific, catalog no. ER2191) and ApaI (NEB, catalog no. 50-811-952); V_K_ fragments were double digested with restriction enzymes BsmI (NEB, catalog no. R0134L) and BsiWI (NEB, catalog no. R3553L). Following double digestion, V_K_ library was generated by ligating 3.5 µg of V_K_ inserts into 10 µg of double digested phagemid vector LVEC22995 (Sanofi proprietary) at a phagemid:insert ratio of 1:4 using T4 DNA ligase (NEB, catalog no. M0202M) at 16 °C overnight in 1 ml reaction volume. Post-ligation, reaction mixture was column purified (Macherey–Nagel, catalog no. 740609) in a final volume of 15 µl, and transformation was carried out by electroporating (Bio-Rad MicroPulser, catalog no. 1652100) 2 µl of ligated mix into 50 µl of TG1 electrocompetent cells (Lucigen Corp., catalog no. 605022) in a 0.1 cm cuvette (Biorad, catalog no. 1652083). Immediately after electroporation, cells were recovered in 1 ml SOC medium for 1 hr. at 37 °C in a shaking incubator and plated overnight on to a large 2YT-Agar square bioassay plate containing 100 µg/ml ampicillin and 2% glucose (Teknova, catalog no. Y6292).

**Table 2:** Primers used for generating Fab expressing phage display libraries. Degenerate nucleotides were used in few primers according to the IUPAC nomenclature (R = A/G, K = G/T, S = C/G, Y = C/T).

RT primers	
IGHG1,2 A,2B,2 C	CTGGACAGGGATCCAGAGTTCCA
IGHG3	CTGGACAGGGCTCCATAGTTCCA
IgK	GGGTGAAGTTGATGTCTTGTGAGTGGC
PCR1 primers for variable-kappa chain amplification	
VKappa1	GCCACCATGGACATGAGG
VKappa2	GCCACCATGGGGTCCCAG
VKappa3	GCCACCATGGTGTCCCCGTT
VKappa4	GCCACCATGGAAACCCCA
VKappa5	GCCACCATGGTGTTGCAG
VKappa6	GCCACCATGGAAGCCCCA
VKappa7	GCCACCATGGACATGAGA
VKappa8	GCCACCATGGAACCATGG
PCR1 primers for variable-heavy chain amplification	
VH1	GCCACCATGGACTGGACC
VH2	GCCACCATGGACTGCACC
VH3	GCCACCATGGACTGGATTTGGAGGG
VH4	GCCACCATGGARTTGGGG
VH5	GCCACCATGGAGTTTGGG
VH6	GCCACCATGGAGTTKGGACTGAGC
VH7	GCCACCATGGAGTTTTGGCT
VH8	GCCACCATGGAATTTGGGC
VH9	GCCACCATGGAACTGGGG
PCR1 reverse primers are the same as used for RT reaction	
PCR2 forward primers for variable-kappa chain amplification	
ForIGKV1-6	GCCATCCAGATGACCCAGTCTCC
ForIGKV1-8	GCCATCCGGATGACCCAGTCTCC
ForIGKV1-17	GACATCCAGATGACCCAGTCTCC
ForIGKV2-24	GATATTGTGATGACCCAGACTCC
ForIGKV3-20	GAAATTGTGTTGACGCAGTCTCC
ForIGKV3D-7	GAAATTGTAATGACACAGTCTCC
ForIGKV4-1	GACATCGTGATGACCCAGTCTCC
ForIGKV6D-41	GATGTTGTGATGACACAGTCTCC
PCR2 forward primers for variable-heavy chain amplification	
ForIGHV3-A	GARGTGCAGCTGKTGGAGTCTGG
ForIGHV3-33	CAGGTGCAGCTGGTGGAGTCTGG
ForIGHV4-A	CAGSTGCAGCTGCAGGAGTCTGGCCCAGGACTG
ForIGHV4-34	CAGGTGCAGCTACAGCAGTGGGG
ForIGHV6-1	CAGGTACAGCTGCAGCAGTCAGG
ForIGHV1-A	CARRTGCAGCTGGTGCAGTCTGG
ForIGHV5-51	GAGGTGCAGCTGGTGCAGTCTGG
ForIGHV1-B	CAGGTYCAGCTGGTRCAGTCTGG
PCR2 reverse primers for variable-kappa chain amplification	
revKJ1	TTTGATTTCCACCTTGGTCCC
revKJ2	TTTGATCTCCAGCTTGGTCCC
revKJ3	TTTGATATCCACTTTGGTCCCAG
revKJ4	TTTGATCTCCACCTTGGTCCC
revKJ5	TTTAATCTCCAGTCGTGTCCCTT
PCR2 reverse primers for variable-heavy chain amplification	
revHJ2	TGAGGAGACAGTGACCAGGGT
revHJ3	TGAAGAGACGGTGACCATTGTC
revHJ4	TGAGGAGACGGTGACCAGG
revHJ6	TGAGGAGACGGTGACCGTG

In parallel, titration was performed to evaluate the transformation efficiency of the TG1 cells. Next day, cells were scrapped from the square plates and a maxiprep was performed to obtain phagemid to clone V_H_ inserts, following similar procedure as described for V_K_ insertion and transformation. For packaging the library into phages, glycerol stocks from bacteria transformed with phagemids containing the V_H_ and V_K_ inserts were inoculated at a cell density 100 times higher than the size of library. Large scale culture was inoculated at an OD600 = 0.1 and was grown at 37 °C and 250 RPM to a cell density of OD600 = 0.5. After that, M13K07 helper phages were added at a MOI = 20 and infection was performed without agitation for 30 min at 37 °C. Cells were then centrifuged at 3000 g for 15 min to eliminate glucose that acts as repressor. Then cells were suspended in 2YT media containing ampicillin (100 µg/ml) and kanamycin (50 µg/ml) and grown overnight in a shaking incubator (250 RPM) at 30 °C. Next day, cells were centrifuged at 3000 g for 15 min to remove any bacteria and the supernatant was filtered with a 0.2-micron filter. A solution of 20% PEG8000 in 2.5 M NaCl (100 ml) was then added to the supernatant at a ratio of 1:5 and kept on ice for one hour with regular shaking every 15 min. The PEG precipitated supernatant was centrifuged for one hour at 10,000 rpm at 4 °C. The pellet was then suspended in 8 ml of PBS and 2 ml of PEG/NaCl and was again centrifuged for one hour at 10,000 rpm at 4 °C. Lastly, phage pellet was suspended in 1 ml of PBS and titration was performed by infecting TG1 cells grown to exponential phase (OD600 = 0.5) with limiting dilutions of phage particles.

### Panning of libraries

The selection for target-specific antibodies was performed by panning AgSC and TBC immune-libraries on biotinylated antigen C or D conjugated to streptavidin beads. Briefly, 110 µl of streptavidin beads were conjugated with 200 nM biotinylated antigen and control antigen-biotin, each, in 500 µl of PBS-BSA-1% (PBSB-1%), for 30 min at RT. Beads were then centrifuged at 3000 g for 5 min, washed twice with 500 µl PBST-0.1% and finally suspended in 500 µl of PBSB-1%. Non-specific phage depletions using beads alone or with control antigen, and positive selections using beads conjugated with target antigens, were carried on the KingFisherTM ml instrument (Thermo Scientific, catalog no. 5400050). A 10^10^ phage titer of AgSC and TBC immune-libraries, which accounts for 100 times higher number of phage particles than library size was diluted in 500 µl of PBSB-1% and the libraries were suspended into first well. The subsequent wells included beads alone, beads with control antigen and target antigen conjugated to beads. Phages from each well were transferred to subsequent wells after incubation for 30 min each. Further, phages bound to target antigen-beads complex were washed five times with 500 µl PBST-0.1% and twice with PBS. Thereafter, phages were eluted with 500 µl fresh 100 mM TEA and neutralized with 250 µl 1 M Tris-HCl (pH 8). Output phages obtained after elution were incubated without shaking for 30 min at 37 ˚C with 5 ml of TG1 cells (OD 600 = 0.5) and thereafter for another 30 min in a shaking incubator (150 RPM) at 37 ˚C. Post-infection, cells were centrifuged at 3000 g and the pellet was spread on a square bioassay plate. Titrations were performed on post-depletion and selection bio-panned phages by performing 1/10th dilutions of phages and thereafter infecting TG1 cells. Glycerol stock from round-one output was used to obtain phages for selection in round-two by infecting cells at OD600 = 0.5 and infecting M13K07 phages at a MOI = 20 and following similar selection parameters as described for round-one.

### Screening on antigens

Phage particles from individual colonies were screened for binding with antigen C or D. To evaluate binding, ELISA was performed on streptavidin coated plates (Thermo Scientific, catalog no. 436014), by first blocking wells with PBS-BSA-3% (300 µl/well) overnight at 4 °C and then adsorbing biotinylated target antigen or control antigen (1 µg/ml, 50 µl/well) for 1 h at RT. Wells were washed thrice with 300 µl PBST-0.1% and phage supernatants (50 µl/well) from individual colonies, previously blocked with PBS-BSA-3% were added to each well and incubated for 1 h at RT. Phage-bound wells were then washed and anti-M13 antibody (0.2 µg/ml, 50 µl/well) labelled with europium was added for 1 hr at RT. Finally, wells were washed thrice with PBST-0.1%, developed with Delfia enhancement solution (Perkin Elmer, catalog no. 4001-0010) and read at 615 nm in an Envision plate reader.

Binding with the target antigen was also tested through a cell-based assay, wherein, phage supernatants (50 µl) from individual colonies as used for ELISA, were incubated with wild type CHO cells and CHO cells expressing antigen D on the cell surface (30,000 cells/well) for 1 hr at RT. Cells were then washed three times with PBS-BSA-1% and further incubated with 50 µl/well of pre-conjugated mouse anti-M13 antibody (1:500 dilution) and Alexa-647 labelled anti-mouse antibody (1:200 dilution) (Invitrogen, catalog no. A28181) in dark at RT for 1 hr. Cells were then washed thrice with PBS-BSA-1%, suspended in 100 µl of PBS-BSA-1% and acquired in an iQue flow cytometry system (Sartorius) to evaluate binding of individual samples. The data was analyzed using Forcyte software^43^. Irrelevant Fab expressing negative control phage particles were also used as controls.

### Sequence analysis of antigen-specific clones

Sanger sequencing was performed on all antigen-binding clones. Antibody sequence analysis was carried out using an in-house wrapper bioinformatics tool based on IgBLAST and excel macros, which allowed the annotation of gene families and identification of unique clones based on different regions of antibodies such as frameworks and CDRs. Frameworks 1 and 4 were not included in the analysis due to the use of degenerate primers during PCR. We concatenated all the six CDRs from V_H_ and V_K_ protein sequences of antibodies from AgSC, TBC libraries and sequences obtained from single B-cells after antigen selection. All phylogenetic tree analyses of antibody clones were done using Neighbor-Joining method with Juke–Cantor distance model as implemented in CLC Main Workbench 8.1 (Qiagen).

### Functional assay to identify receptor antagonist antibodies against antigen C

Whole blood or buffy coat was mixed with 4 ml PBS and layered on 15 ml Ficoll-Paque PLUS (GE Healthcare, catalog no. 17144003) in 50 ml centrifuge tubes and centrifugation was performed at 2000 rpm for 20 min at RT. Peripheral blood mononuclear cell (PBMC) layer was collected and washed with PBS (Gibco, catalog no. 14190144) and then treated with ACK lysis buffer (Gibco, catalog no. A1049201) to remove residual red blood cells. The cells were washed twice with FACS buffer containing 2% bovine serum albumin (Sigma, catalog no. A7949) and 1 mM EDTA in PBS, and then resuspended in the assay media containing RPMI (Gibco, catalog no. 22400089), 10% fetal bovine serum (Thermo Fisher Scientific, catalog no. 10082147) and 1% Penicillin/Streptomycin (CORNING, catalog no. 30001CI).

Prior to the functional assay, each well of the 96-well assay plates (Greiner Bio-One, catalog no. 781080) were seeded with 20,000 parental CHO cells in control wells and CHO cells expressing antigen C in assay wells in 100 μl of the assay medium for 4 h. Then, 100 μl of PBMCs (300,000/well) that were pre-incubated for 15 min in the presence or absence of monoclonal antibodies were seeded into each well. Finally, 50 μl of cytokine IL-15 at a final concentration of 2 ng/ml was added into each well and the cells were incubated for 2 days at 37 °C before collections of supernatants. The read-out for the receptor-antagonist functional assay was the amount of GM-CSF produced by PBMCs when cocultured with CHO cells with or without the expression of antigen C and was determined by ELISA using a human GM-CSF ELISA kit (R&D Systems catalog no. DY215).

### Functional assay using recombinant IgGs against antigen D in a high-throughput reporter assay

NFkB-Luc2P/U2OS reporter cells (Promega GloResponse™; Promega Corporation) were grown in McCoy’s 5 A medium (Thermo Fisher Scientific, catalog no. 16600082) with 10% fetal bovine serum (Thermo Fisher Scientific, catalog no. 10082147) and 200 mg/ml Hygromycin B (Thermo Fisher Scientific, catalog no. 10687010) as per manufacturer recommendations. Reporter cells were resuspended in McCoy’s 5 A supplemented with 1% fetal bovine serum before seeding into assay plates. High-throughput functional assay run was performed on an automated GNF (Genomics Institute of the Novartis Research Foundation) screening system, equipped with a Stäubli TX90L robotic arm (Stäubli Corporation) serving an integrated Envision plate reader (Perkin Elmer Inc.), a Bravo liquid handling platform (Agilent Technologies Inc.), two GNF Model I washers/dispensers, and two Thermo Fisher incubators (Forma Environmental Chamber, model 4933, Thermo Fisher Scientific).

Reporter cells were seeded at 40,000 cells per well in 12.5 μl of assay medium into 384-well assay plates (Greiner Bio-One, catalog no. 781080) for screening functional activity of antibodies against each antigen. In parallel, 384-well master plates were created on the Agilent Bravo to make 9-point or 18-point serial dilution of antibody (or test) samples and positive control. Sample solutions (12.5 µl) from the master plates were then added into wells of three replicate assay plates and incubated for 4 h at 37 °C and equilibrated for 15 min at RT. 50 μl/well of Bio-Glo detection reagent (Promega Corporation, catalog no. G7940) was added to the assay plates using the GNF dispenser and Luminescence was measured on the Envision after for 5–10 min using the US luminescence aperture and a readout time of 0.1 sec/well. Raw data files were imported from the Envision into Genedata Screener (version 16.0.2, Genedata). A custom Knime pipeline (Knime AG) was used to add the well metadata in a cmt file. Negative and positive controls were utilized as central and scale references to normalize and scale the data. Plate-based RZ’ factors were calculated in Screener and plates with RZ’ < 0.5 were masked and excluded from further analysis. Valid IC50 values were reported as qAC50 values by curve fitting in the Screener SmartFit algorithm.

### Next-generation sequencing of V_H_ genes from AgSC and TBC library panning outputs against antigen D

Bacterial pools from panning output of AgSC and TBC libraries against antigen D were used to amplify the V_H_ genes for NGS. The glycerol stocks of bacteria infected with the panning outputs from AgSC, and TBC libraries (1.5 ml each) were used to extract the phagemids with a midi-prep plasmid isolation kit (Qiagen, catalog no. 12943). V_H_ genes were amplified with primers specific for the signal peptide and CH1 regions. Briefly, in a 50 µl reaction volume, 1 µl of phagemid was mixed with 1 µl 10 mM dNTPs (Invitrogen, catalog no. 18427088), 1 µl each of 10 µM forward and reverse primers, 0.5 µl of Q5 hot-start polymerase (NEB, catalog no. M0493L), 10 µl Q5 reaction buffer and 35.5 µl nuclease-free water; PCR was performed at 98 °C for 30 sec (initial denaturation) followed by 25 cycles of 98 °C for 10 sec (cycle denaturation), 72 °C for 30 sec (primer annealing), 72 °C for 30 sec (primer extension), and final extension at 72 °C for 5 min. The amplified V_H_ gene fragments were separated on a 1% agarose gel followed by gel purification (Macherey–Nagel, catalog no. 740609). The purified V_H_ gene fragments were sequenced by the Amplicon-EZ service (Genewiz), which provides a sequencing depth of 50,000 paired reads with 2 × 250 bp length through an Illumina Mi-Seq sequencer.

### Expression and purification of recombinant Fabs from functional clones against antigen C and D from AgSC and TBC libraries

The cloning and expression of Fabs was performed using our in-house high-throughput method^44^ The variable regions genes of functional antibodies against antigens C and D from the AgSC and TBC libraries were commercially synthesized as gBlock^®^ DNA fragments (Integrated DNA Technologies) and then cloned into the expression vector pTT5 by the In-Fusion^®^ cloning method (Takara Bio Inc.). The expression and purification of Fabs was performed on a fully automated platform of mammalian cell overexpression system, Protein Expression and Purification Platform (PEPP; GNF). The cloned plasmid vectors (34 µg) were transfected into 3.2 × 10^6^ Expi293F cell (Thermo Fisher Scientific, catalog no. A14527) using the Expifectamine 293 transfection reagent (Thermo Fisher Scientific, catalog no. A14525). The cells were then incubated for 5 days at 37 °C with 8% CO_2_. The supernatants were harvested and Fabs were isolated by gravity flow by the MabSelect SuRe protein A resin (GE Healthcare, catalog no. 17-5438-02) followed by desalting by Nap10 Sephadex (GE Healthcare, catalog no. 17-0854-02) and eluting with 1X DPBS (Thermo Fisher Scientific, catalog no. 14190136).

### Measurement of the melting temperatures (T_M_) of Fabs to establish thermal stability

Nano Differential Scanning Fluorimetry (NanoDSF) was used to measure the melting temperature (T_M_) of the Fabs using the biologics stability screening platform Uncle^®^ (Unchained labs). The method measures the change in intrinsic fluorescence of proteins to monitor protein unfolding with increasing temperature. The samples were normalized to 0.5 mg/ml in PBS pH 7.2. The intrinsic fluorescence was monitored over a thermal ramp from 20 °C to 95 °C with a ramp rate of 1 °C/min. The protein solution was excited using a 266 nm wavelength light source and the fluorescence emission of tyrosine and tryptophan at 330 nm and 350 nm, respectively, were measured. The emission maxima and the intensity of tyrosine and tryptophan are highly dependent on their immediate environment and can change as the protein unfolds during thermal denaturation. Monitoring the change in fluorescence intensity from 250 to 700 nm as a function of temperature yielded a sigmoid shaped curve that represents the unfolding transition of the protein. The midpoint of the sigmoid curve represented the melting temperature (TM) and was obtained by the inflection point on the first derivative of the curve.

### Affinity measurement of Fabs from functional clones against antigen C and D from AgSC and TBC libraries

Recombinant Fabs of functional clones were tested for affinity against antigen C and D on Carterra LSA instrument (Carterra) utilizing an in-house produced Fab Capture HC200M Chip (Carterra). The LSA single flow cell was used to prepare the lawn, which involved (1) activating the chip with a 7-min injection of 1:1:1 v/v/v mixture of 0.4 M EDC + 0.1 M sulfo-NHS + 0.1 M MES pH 5.5, (2) coupling 5 µg/ml Goat Anti-Human IgG, F(ab’)2 specific antibody (Jackson Immunoresearch, 109-005-097) in 10 mM sodium acetate pH 4.5 for 10 min, and (3) blocking excess reactive esters with a 8-min injection of 1 M ethanolamine HCl pH 8.5. Recombinant Fab samples were normalized to 100 µg/ml and then diluted in HBS-EP buffer (In-house, 0.01 M HEPES pH 7.4, 0.15 M NaCl, 3 mM EDTA, 0.005% v/v Tween20) to a final concentration of 5 µg/ml for capture. Antigens C or D were diluted to a concentration of 500 nM in HBS-EP; a 12-point, 2-fold dilution curve was then generated via serial dilution of the initial concentration. These were loaded into the instrument and the binding kinetics was performed as follows; 10-minute antibody capture, followed by antigen flow settings of 1 minute equilibration, 5-minute association, and 20-minute disassociation using HBS-EP as the running buffer. Data was analyzed using the kinetics software (Carterra) with a minimal Rmax threshold of 10.

### Statistics and reproducibility

The statistical tests and sample sizes have been referred along with the figure legends of each figure. The statistical significance of phage ELISA between clones from AgSC and TBC libraries was determined using two-tailed unpaired t-test. The p-values for expression levels, thermal stability, and affinity of functional antibodies from AgSC and TBC libraries against antigens C and D was determined using non-parametric Mann Whitney test. The reproducibility of our bulk selection method for enrichment of antigen-specific B cells was ensured by replicating the process on five different targets. Phage libraries from antigen-specific and total B cells were evaluated for 399 and 127 unique antibody clones for the two target antigens, respectively.

### Reporting summary

Further information on research design is available in the Nature Research Reporting Summary linked to this article.

## Supplementary information

Supplementary Figures

Description of Additional Supplementary Files

Supplementary Data

Reporting Summary

## Data Availability

The antibody sequence datasets generated in the current study are available from Sanofi, but restrictions apply to the availability of these data, and they are not publicly available. The data are however available from the authors upon reasonable request and with permission of Sanofi. The numerical source data for all applicable graphs is provided in the excel file named “Supplementary Data”.
