# Supplementary material for: Honing-in antigen-specific cells during antibody discovery: a user-friendly process to mine a deeper repertoire
Source: Commun Biol. 2022 Oct 30;5:1157. doi: 10.1038/s42003-022-04129-7 (PMC9618561; doi:10.1038/s42003-022-04129-7)
Supplement: Supplementary file 1 — Supplementary Figures [file 42003_2022_4129_MOESM1_ESM.pdf]

Supplementary Figure 1.

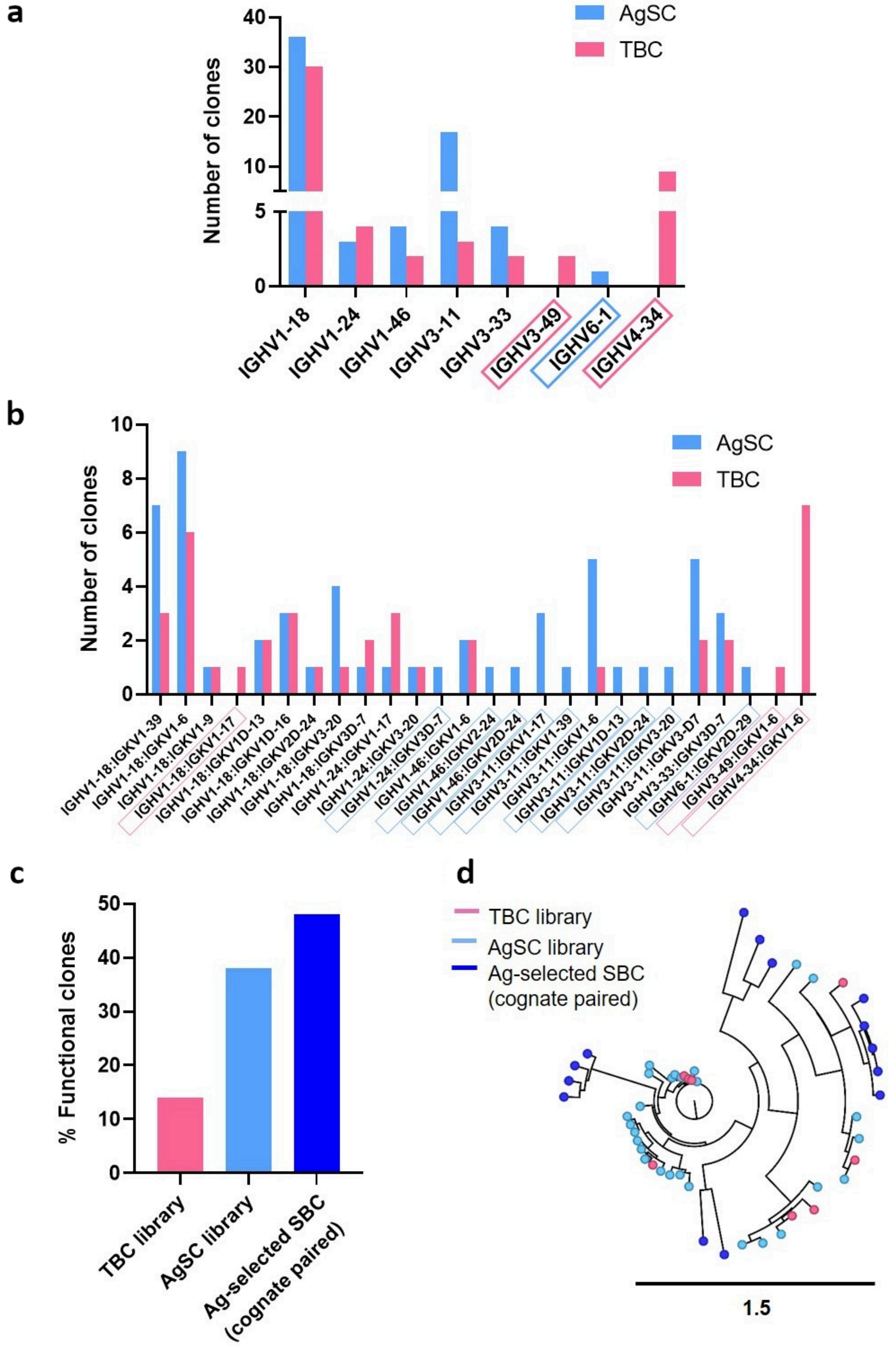

Supplementary figure 1. Comparative analysis of antibody clones from AgSC and TBC libraries against antigen D. (a) Graph shows heavy chain V-gene usage by antigen reactive unique clones from AgSC and TBC libraries. Annotation is based on IgBLAST classification. Blue boxes represent V_H-genes exclusive to clones from AgSC library and pink represents those exclusive to clones from TBC library. (b) V_H and V_K pairing combinations in clones from the two libraries are presented in bar graph. Blue boxes represent V_H-V_K combinations exclusive to clones from AgSC library and pink represents those exclusive to clones from TBC library. (c) Percent functional antibodies were identified from TBC library (8 of total 56 clones or 14%), AgSC library (27 of total 71 clones or 38%), and cognate paired Ag-selected single B cells (15 of 31 clones or 48%). (d) Radial phylogenetic mapping of functional clones derived from AgSC and TBC libraries, and cognate paired Ag-selected single B cells illustrates the diversity of clones from these sources. The scale represents Juke-Cantor distance model as explained in the methods section. Cognate chain paired clones showed the greatest diversity followed by phage clones from AgSC and then by TBC libraries.

Supplementary Figure 2.

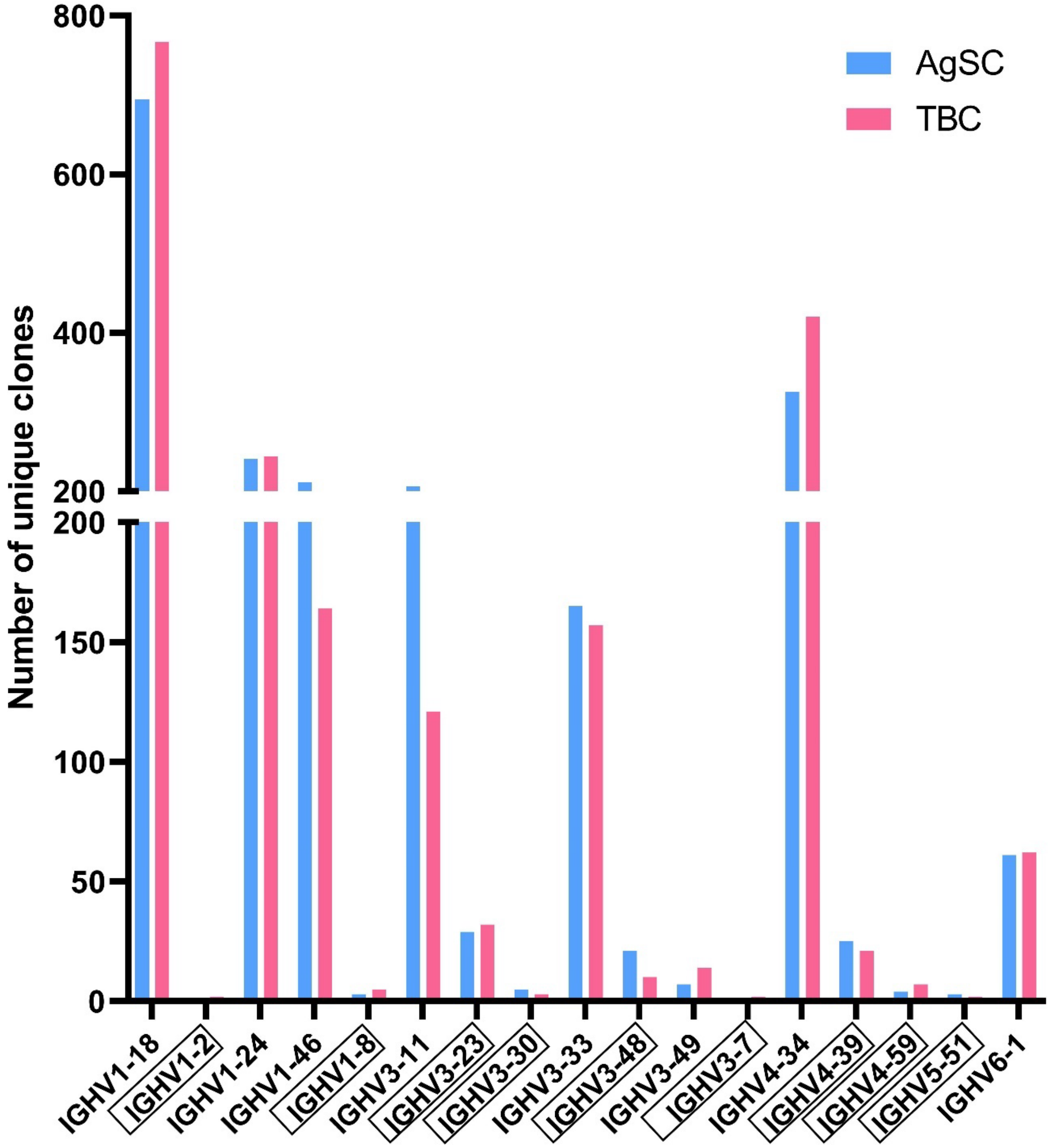

Supplementary figure 2. Next generation sequencing of V_H-genes from phage panning clone pools of AgSC and TBC libraries against antigen D. Unique clones from AgSC (N = 2000) and TBC (N = 2033) libraries are plotted as number of clones with the same V_H germline gene. Clones with at least two or more read counts were considered for analysis. The gene family *IGHV3-11*

displays high usage among AgSC clones as observed in the sanger sequencing data. Gene families marked with black boxes are those identified by NGS and were not observed with sanger sequencing. The clones identified by NGS were not tested for antigen specificity. N = unique antibody clones.

Supplementary Figure 3.

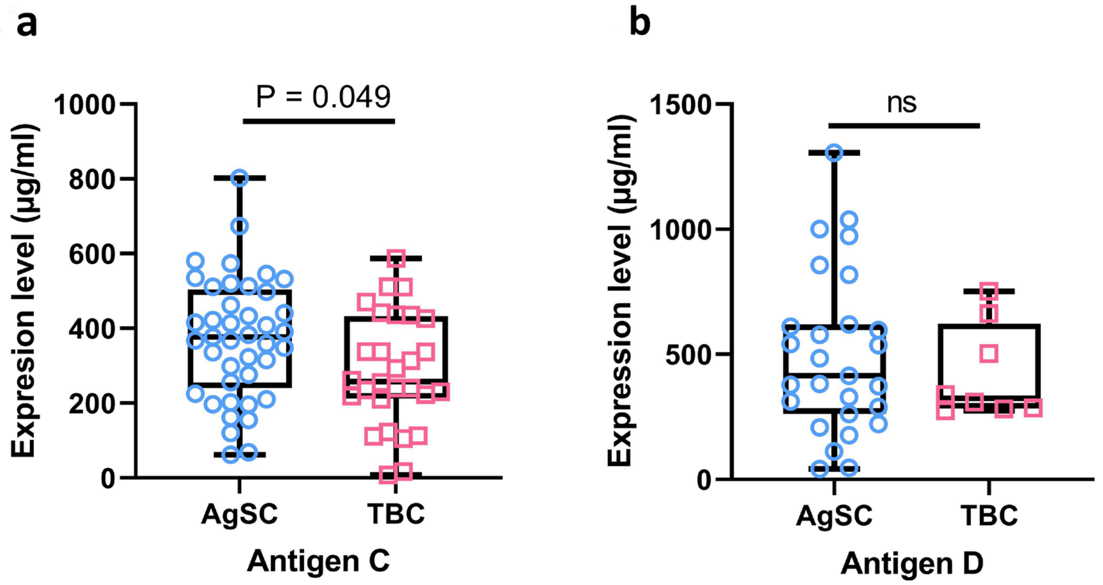

Supplementary figure 3. Box plots of expression levels of recombinant Fabs of functional clones against antigens C and D from AgSC and TBC libraries. (a) Expression level of Fabs from equal volumes of culture supernatant for functional clones against antigen C from AgSC (N = 41) and TBC (N = 28) libraries. (b) Expression level of Fabs from equal culture volumes for functional clones against antigen D from AgSC (N = 27) and TBC (N = 8) libraries. The boxes display upper and lower quartiles with the median value. Whiskers display the minimum and maximum expression level (µg/ml) of recombinant Fabs. Individual circles and squares represent expression levels of each clone expressed as Fab. Statistical analysis was performed using non-parametric Mann Whitney test. N = functional antibody clones.

Supplementary Figure 4.

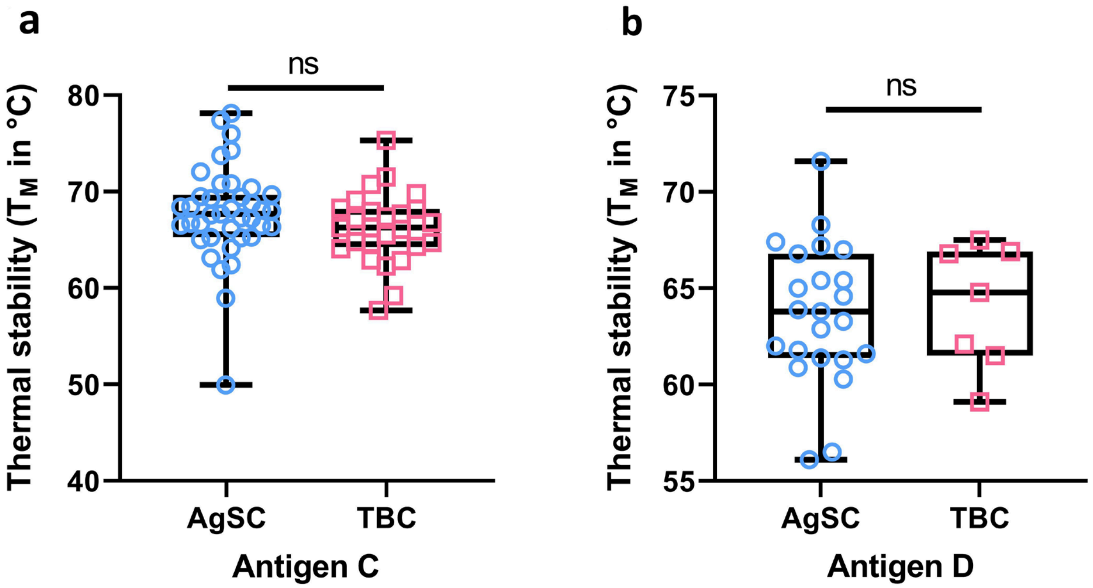

Supplementary figure 4. Thermal stability of recombinant Fabs of functional clones against antigens C and D from AgSC and TBC libraries measured as melting temperature (T_M). (a) Comparative T_M of recombinant Fabs of functional clones from AgSC (N = 39) and TBC (N = 26) libraries against antigen C. (b) Comparative T_M of recombinant Fabs of functional clones from AgSC (N = 23) and TBC (N = 7) libraries against antigen D. The boxes display upper and lower quartiles with the median value. Whiskers display the minimum and maximum melting temperature values (°C) of recombinant Fabs. Individual circles and squares represent melting temperature values of each clone expressed as Fab. Statistical analysis was performed using non-parametric Mann Whitney test. N = functional antibody clones.

Supplementary Figure 5.

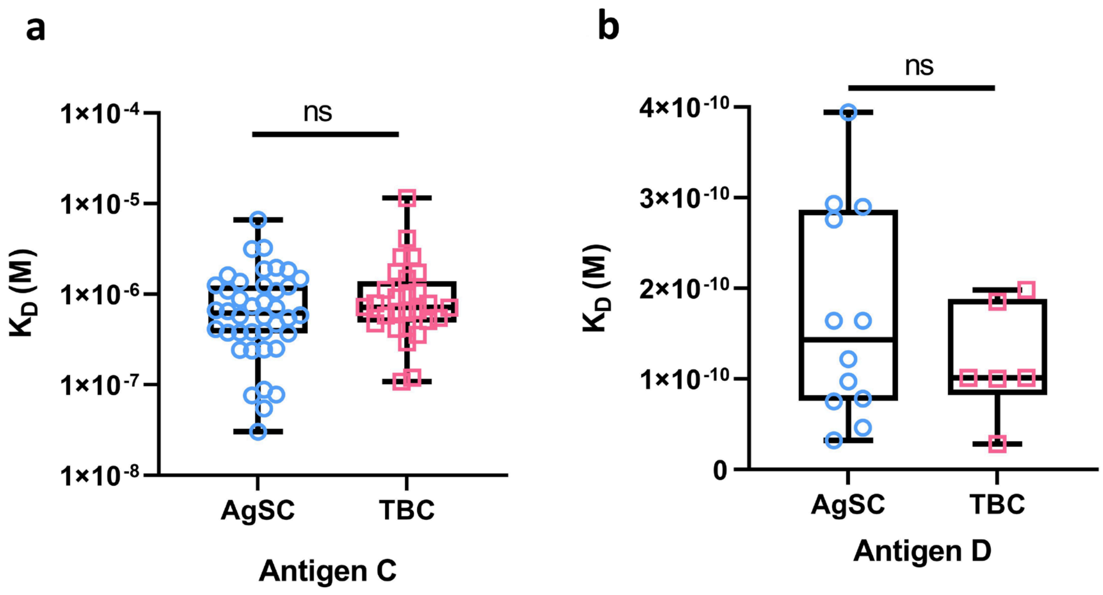

Supplementary figure 5. Affinity measurement of recombinant Fabs by SPR of functional clones obtained from AgSC and TBC libraries against antigens C and D. (a) Graph shows affinity measurement of recombinant Fabs of functional clones from AgSC (N = 40) and TBC (N = 28) libraries against antigen C. (b) Affinity measurement of recombinant Fabs of functional clones from AgSC (N = 12) and TBC (N = 6) libraries against antigen D. The boxes display upper and lower quartiles with the median value. Whiskers display the minimum and maximum affinity values. Individual circles and squares represent affinity of each clone expressed as Fab. Statistical analysis was performed using non-parametric Mann Whitney test. N = functional antibody clones.
