# Supplementary material for: Honing-in antigen-specific cells during antibody discovery: a user-friendly process to mine a deeper repertoire
Source: Commun Biol. 2022 Oct 30;5:1157. doi: 10.1038/s42003-022-04129-7 (PMC9618561; doi:10.1038/s42003-022-04129-7)
Supplement: Supplementary file 4 — Reporting Summary [file 42003_2022_4129_MOESM4_ESM.pdf]

nature research

Corresponding author(s): Ankit Mahendra
Partha S. Chowdhury

Last updated by author(s): Oct 14, 2022

Reporting Summary

Nature Research wishes to improve the reproducibility of the work that we publish. This form provides structure for consistency and transparency in reporting. For further information on Nature Research policies, see our Editorial Policies and the Editorial Policy Checklist.

Statistics

For all statistical analyses, confirm that the following items are present in the figure legend, table legend, main text, or Methods section.

n/a	Confirmed	
☐	☒	The exact sample size (*n*) for each experimental group/condition, given as a discrete number and unit of measurement
☐	☒	A statement on whether measurements were taken from distinct samples or whether the same sample was measured repeatedly
☐	☒	The statistical test(s) used AND whether they are one- or two-sided<br>*Only common tests should be described solely by name; describe more complex techniques in the Methods section.*
☒	☐	A description of all covariates tested
☒	☐	A description of any assumptions or corrections, such as tests of normality and adjustment for multiple comparisons
☐	☒	A full description of the statistical parameters including central tendency (e.g. means) or other basic estimates (e.g. regression coefficient) AND variation (e.g. standard deviation) or associated estimates of uncertainty (e.g. confidence intervals)
☒	☐	For null hypothesis testing, the test statistic (e.g. *F*, *t*, *r*) with confidence intervals, effect sizes, degrees of freedom and *P* value noted<br>*Give P values as exact values whenever suitable.*
☒	☐	For Bayesian analysis, information on the choice of priors and Markov chain Monte Carlo settings
☒	☐	For hierarchical and complex designs, identification of the appropriate level for tests and full reporting of outcomes
☒	☐	Estimates of effect sizes (e.g. Cohen's *d*, Pearson's *r*), indicating how they were calculated

Our web collection on statistics for biologists contains articles on many of the points above.

Software and code

Policy information about availability of computer code

Data collection	Forcyte software was used to analyze Flow cytometry data from iQue.<br>Antibody sequence analysis was performed using in-house bioinformatic tool based on IgBLAST.<br>CLC Main Workbench 8.1 (Qiagen) was used for phylogenetic tree analysis.<br>Functional data was analyzed by Genedata Screener (version 16.0.2, Genedata).<br>Metadata of wells in the functional assay was added using custom Knime pipeline (Knime AG).<br>Curve fitting to obtain IC50 values was done using the Screener SmartFit algorithm (version 16.0.2, Genedata).
Data analysis	Flow cytometry data from iQue was analyzed using Forcyte software. Antibody sequence analysis was carried out using an in-house wrapper bioinformatics tool based on IgBLAST and excel macros. All phylogenetic tree analyses of antibody clones were done using Neighbor-Joining method with Juke-Cantor distance model as implemented in CLC Main Workbench 8.1 (Qiagen). Functional assay was analyzed by importing raw data files from the Envision into Genedata Screener (version 16.0.2, Genedata). A custom Knime pipeline (Knime AG) was used to add the well metadata in a cmt file. Negative and positive controls were utilized as central and scale references to normalize and scale the data. Plate-based RZ' factors were calculated in Screener and plates with RZ' <0.5 were masked and excluded from further analysis. Valid IC50 values were reported as qAC50 values by curve fitting in the Screener SmartFit algorithm.

For manuscripts utilizing custom algorithms or software that are central to the research but not yet described in published literature, software must be made available to editors and reviewers. We strongly encourage code deposition in a community repository (e.g. GitHub). See the Nature Research guidelines for submitting code & software for further information.

Data

Policy information about availability of data

All manuscripts must include a data availability statement. This statement should provide the following information, where applicable:

- Accession codes, unique identifiers, or web links for publicly available datasets
- A list of figures that have associated raw data
- A description of any restrictions on data availability

The antibody sequence datasets generated in the current study are available from Sanofi, but restrictions apply to the availability of these data, and they are not publicly available. The data are however available from the authors upon reasonable request and with permission of Sanofi. The numerical source data for all applicable graphs is provided in the excel file named "Supplementary Data".

Field-specific reporting

Please select the one below that is the best fit for your research. If you are not sure, read the appropriate sections before making your selection.

☒ Life sciences ☐ Behavioural & social sciences ☐ Ecological, evolutionary & environmental sciences

For a reference copy of the document with all sections, see nature.com/documents/nr-reporting-summary-flat.pdf

Life sciences study design

All studies must disclose on these points even when the disclosure is negative.

Sample size	We did not perform a sample size calculation method, however, we have ensured that a large data set was evaluated to derive to the conclusions observed in our study. To determine the specificity of B cells isolated through our bulk enrichment process, we tested the method on five different target antigens. A total of 288 individual IgGs from the antigen-selected group and 96 individual IgGs from the input and flow-through B-cell groups were expressed and tested for gene recovery, expression, and target binding.<br>To derive conclusions for our phage library experiments, we examined 399 and 127 unique antibody clones for the two target antigens, respectively.
Data exclusions	No data was excluded from the analysis
Replication	The reproducibility of our bulk selection method for enrichment of antigen-specific B cells was ensured by replicating the process on five different targets. Phage libraries from antigen-specific and total B cells were evaluated for two different target antigens.
Randomization	Not relevant to our study because organisms and patients are not involved in the evaluation of antibodies.
Blinding	Blinding was not necessary because group allocation was not required during data collection/analysis.

Reporting for specific materials, systems and methods

We require information from authors about some types of materials, experimental systems and methods used in many studies. Here, indicate whether each material, system or method listed is relevant to your study. If you are not sure if a list item applies to your research, read the appropriate section before selecting a response.

Materials & experimental systems

n/a	Involved in the study	
☐	☒	Antibodies
☐	☒	Eukaryotic cell lines
☒	☐	Palaeontology and archaeology
☐	☒	Animals and other organisms
☒	☐	Human research participants
☒	☐	Clinical data
☒	☐	Dual use research of concern

Methods

n/a	Involved in the study	
☒	☐	ChIP-seq
☐	☒	Flow cytometry
☒	☐	MRI-based neuroimaging

Antibodies

Antibodies used	anti-mouse IgD monoclonal antibody (clone 11-26c, Fisher Scientific, catalog no. 50-112-8799)<br>anti-mouse IgM micro-beads (Miltenyi Biotec, catalog no. 130-047-301)<br>anti-mouse IgG (H+L) (Jackson ImmunoResearch catalog no. 115-035-166)<br>Alexa-647 labeled anti-mouse antibody (Invitrogen, catalog no. A28181)
Validation	anti-mouse IgD monoclonal antibody (Applications: Flow Cytometry, Immunohistochemistry-Frozen Sections,

Validation	Immunohistochemistry-Paraffin Sections, Immunoprecipitation, Separation; RRID- AB_2631189); Citation:J Exp Med. 2003;198:1157-69<br><br>anti-mouse IgM micro-beads (Applications: isolation of lymphocyte subsets, for the isolation of murine primordial germ cells, isolation of follicular dendritic cells from human tonsils); Citation: Blood 2002; 2905-2912<br><br>anti-mouse IgG (H+L) (Application: Immunohisto/cytochemistry, ELISA, Western blot; RRID: AB_2338511); Citation: JBC 2021;101405<br><br>Alexa-647 labeled anti-mouse antibody (Application: Immunocytochemistry, Flow cytometry; RRID: AB_2536165); Citation: Nat. Comm. 2018; 10.1038/s41467-018-03856-y.

Eukaryotic cell lines

Policy information about cell lines

Cell line source(s)	Expi293F (Fisher Scientific, catalog no. A14527)<br>CHO cells (in-house)
Authentication	None of the cell lines used were authenticated.
Mycoplasma contamination	Cell lines were negative for mycoplasma contamination.
Commonly misidentified lines (See ICLAC register)	No commonly misidentified lines were used in the study.

Animals and other organisms

Policy information about studies involving animals; ARRIVE guidelines recommended for reporting animal research

Laboratory animals	Eight to ten-week-old Trianni mice (HHKK) (Trianni, San Francisco, USA) were used in all studies. Both male and female mice were used.
Wild animals	No wild animals were used in the study.
Field-collected samples	No Field-collected samples used in the study.
Ethics oversight	All experiments were approved and conducted according to the guidelines of Sanofi Genzyme Institutional Animal Care and Use Committee.

Note that full information on the approval of the study protocol must also be provided in the manuscript.

Flow Cytometry

Plots

Confirm that:

☐ The axis labels state the marker and fluorochrome used (e.g. CD4-FITC).

☐ The axis scales are clearly visible. Include numbers along axes only for bottom left plot of group (a 'group' is an analysis of identical markers).

☐ All plots are contour plots with outliers or pseudocolor plots.

☐ A numerical value for number of cells or percentage (with statistics) is provided.

Methodology

Sample preparation	B cells (CD19+, CD19+CD138+ and CD138+) including plasma cells were isolated by immunomagnetic negative selection from total lymphocyte suspension by using EasySep Mouse B-Cell Isolation Kit (Stem Cell Technologies, catalog no. 19854). In brief, lymphocyte suspension was prepared (1 x 108 cells/ml in 0.25 - 2 ml PBS, 0.5% BSA) in 5-ml polystyrene round-bottom tube (12 x 75 mm) (Stem Cell Technologies, catalog no. 38007) and rat serum was added (50 µl/ml of cell suspension). Isolation cocktail containing biotinylated antibodies recognizing specific cell surface markers for non-B cells was added to the sample (50 µl/ml of cell sample) and mixed and incubated for 5 minutes at RT. Magnetic beads (EasySep Streptavidin RapidSpheres 50001) were added to the sample (50 µl/ml of cell suspension), mixed and incubated for 3 minutes at RT. The volume of the sample was made to 2.5 ml by adding PBS, 0.5% BSA. Non-B cells labeled with biotinylated antibodies and streptavidin-coated magnetic particles were discarded by magnetic separation (EasySep Magnet, Stem Cell Technologies, catalog no. 18000) and total B cells were isolated in solution. Cell number was determined using Vi-cell XR cell counter (Beckman Coulter).
Instrument	Single cells were sorted into wells of 96-well plates using BD Influx instrument (BD Biosciences) set with a 70-µM nozzle and 45 PSI pressure.<br>iQue flow cytometry system (Sartorius) was used to evaluate binding of Fab-expressing phage particles on CHO cells expressing antigen.

Software	Flow cytometry data was collected using FACS Diva (BD Biosciences) software for single-cell sorting.<br>The iQue data was analyzed using Forcyte software.
Cell population abundance	The purity of post-sort fraction was determined by recovering paired VH and VK IgG genes from single B cells for all 5 antigens. High efficiency of antibody gene recovery was observed for all 5 antigens , A (86%), B (81%), C (95%), D (94%), E (99%).
Gating strategy	Magnetically enriched IgG+ B cells were labeled with 7-AAD (7-amino actinomycin D) staining solution (ThermoFisher, catalogue no. 00-6993-50) for 5 minutes at RT to exclude dead cells during sorting. Single cells were sorted from the live-cell gate into wells of 96-well plates using BD Influx instrument (BD Biosciences).

☐ Tick this box to confirm that a figure exemplifying the gating strategy is provided in the Supplementary Information.
